# The role of host mobility in the transmission and spread of *Echinococcus granulosus*: A Chile-based mathematical modeling approach

**DOI:** 10.1371/journal.pntd.0012948

**Published:** 2025-04-14

**Authors:** Richard Lagos, Juan Pablo Gutiérrez-Jara, Beatriz Cancino-Faure, Leidy Yissedt Lara-Díaz, Aníbal Coronel

**Affiliations:** 1 Programa de Doctorado en Modelamiento Matemático Aplicado, Facultad de Ciencias Básicas, Universidad Católica del Maule, Talca, Chile; 2 Departamento de Matemática y Física, Universidad de Magallanes, Punta Arenas, Chile; 3 Centro de Investigación de Estudios Avanzados del Maule (CIEAM), Vicerrectoría de Investigación y Postgrado, Universidad Católica del Maule, Talca, Chile; 4 Laboratorio de Microbiología y Parasitología, Departamento de Ciencias Preclínicas, Facultad de Medicina, Universidad Católica del Maule, Talca, Chile; 5 Departamento de Matemática, Física y Estadística, Universidad Católica del Maule, Talca, Chile; 6 Departamento de Ciencias Básicas, Facultad de Ciencias, Universidad del Bío-Bío, Chillán, Chile; IRNASA, CSIC, SPAIN

## Abstract

This paper explores, as a proof-of-concept, the impact of definitive and intermediate host mobility on the transmission and spread of cystic echinococcosis by characterizing disease dynamics using three classical epidemic models: S-E-I-R for the accidental intermediate host, S-E-I for the habitual intermediate host, and S-I-S for the definitive host. The simulations revealed a significant relationship between the mobility of dogs and the increase in infected sheep. Specifically, for each infected dog, there were twice as many infected sheep as in a situation where mobility was not a factor. The initial conditions took into account that the prevalence of the disease in dogs is higher in rural areas than in peri-urban areas, as has been observed in the Magallanes region of Chile. The results of the simulations suggest that mobility can have a role in the propagation of the disease in humans. Furthermore, the sensitivity index on $R_{0}$ indicates that a 10% reduction in the average time spent by peri-urban dogs in urban and rural areas could result in a decrease of approximately 1% in $R_{0}.$ In conclusion, including the host mobility factor allows us to observe that, in general, the number of infected in the domestic cycle of the disease increases, i.e., our mathematical model provides valuable information on the impact of host mobility on the transmission and spread of cystic echinococcosis.

## Author summary

Cystic echinococcosis is a zoonotic disease that has been classified as a neglected tropical disease. In Chile, there are epidemiological situations of high, medium and low endemicity, with an increase in incidence towards the south of the country, where more than 50% of sheep population is concentrated. The disease mainly affects individuals residing in rural settings. The migration of rural human populations, together with small flocks of sheep, to peri-urban areas has led to an expansion in the geographical distribution of the disease. The present study employed mathematical modeling and computer simulations to investigate the impact of dog, sheep and human mobility on the transmission and spread of cystic echinococcosis. Our findings indicate that the number of dogs, sheep and humans infected in the domestic cycle of the disease is higher when considering their mobility between peri-urban, urban, and rural areas. Our model provides insights into the role of host mobility in the transmission and spread of cystic echinococcosis, which could inform strategies for controlling infection in dogs and sheep and reducing human infections.

## Introduction

Cystic echinococcosis (CE) is a zoonotic disease that has been identified on five continents and is currently classified by the World Health Organization as a neglected tropical disease (NTD). The disease is most prevalent in Mediterranean and temperate climates of Asia, Australia, and Latin America [1,2]. It has a high prevalence in Argentina, Uruguay, Peru, and southern Brazil [3]. In Chile, there are epidemiological situations of high, medium, and low endemicity with an increased incidence towards the south of the country. For the year 2022 in the 16 regions of Chile, it is reported that one region presents high endemicity with an incidence rate of 40.7 per 100,000 inhabitants, five regions present medium endemicity with incidence rates between 5.2 and 18.5 per 100,000 inhabitants and 10 regions present incidence rates between 0.3 and 3.0 per 100,000 inhabitants, which is a low endemicity [4]. The Aysén and Magallanes regions, which contain more than 50% of the sheep population, are areas of particular concern due to their high incidence of the disease in humans [1,4,5]. According to the World Health Organization [6], in endemic places, the highest prevalences of human CE are found mainly in rural areas and can reach 5 to 10%, even in the study by Mosbahi et al [7], the prevalence of this disease in children is estimated at 22.1% in Kenia. In Chile, the prevalence of this disease in children is estimated to be between 5 and 10% [8]. In Chile, the disease is attributed exclusively to the cestode *Echinococcus granulosus* (EG) [1]. The dog serves as the definitive host, while the sheep act as an intermediate host, thereby mantaining the life cycle of the parasite. The adult form of the parasite is found in the intestinal villi of the dog, while the larval form is found in the viscera (liver and lungs) of the sheep [3]. In the domestic cycle of EG, sheep ingest parasite eggs found in grass and water. Hydatid cysts develop in the viscera of the host. During slaughter, dogs have the possibility of accessing the viscera of parasitized sheep and feeding on them, becoming infected and developing the adult EG parasite in their intestine. Humans, adults and children (accidental hosts), can become infected with EG by direct contact with a dog that is parasitized with this parasite or by ingesting EG eggs when feeding on vegetables or drinking water that has been contaminated with fecal material from a parasitized dog [6,9–12]. In most human infections, the asymptomatic period can extend for more than ten years, depending on the organ affected, until the hydatid cysts reach a size that causes clinical signs and symptoms [6,13]. This disease produces a severe clinical condition with a high socioeconomic impact since, in adults, it can cause disability and long periods of work absenteeism whose recovery requires resources associated with hospitalization and surgery [14]. CE primarily affects people in rural areas who are involved in sheep farming and who feed viscera from infected slaughtered sheep to their dogs. However, migratory movements from rural to peri-urban areas of human populations with their small flocks of sheep and the practice of feeding viscera to their dogs have increased the geographic distribution of the disease and its potential public health impact [2,15].

In sheep, cysts can disrupt the function of the affected organ and cause slow body growth, with decreased milk, meat, and wool production and a reduced birth rate [16]. However, due to the slow development of cysts, many infected sheep are culled before the cysts cause clinical signs [9,16]. The primary productive activity in the rural zone is sheep farming, which is distinguished by meat, wool, and milk production systems [17]. Additionally, informal sheep meat sales occur at farmer’s market in urban areas [16,18]. In the context of productive development, the peri-urban zone should also be considered as a transitional zone between urban and rural zones [19]. In this way, a dynamic is generated that involves mobility and remains of the hosts between peri-urban, urban and rural areas [17].

In urban and rural areas, factors that have historically been regarded as pivotal to the perpetuation of the EG life cycle are the domestic slaughtering of sheep, the feeding of sheep viscera to dogs, and the irregular deworming of dogs [20–22]. Additionally, there is a clear correlation between the population of wild definitive hosts and the risk of echinococcosis transmission to humans in peri-urban and urban areas that could also be present in the domestic cycle of the disease [23,24]. Therefore, as CE is a complex zoonosis involving various hosts, the regular presence of this disease in a given region affects public health, animal health, and its regional economy and requires integrative tools to improve the processes of epidemiological surveillance, prevention, and control [21].

From 1979 to 2004, an official program to control the transmission of EG in the Magallanes region of Chile resulted in a significant reduction in the prevalence of the disease in dogs, from 70% to 1.8% [25]. However, despite these efforts, the transmission of the disease was not fully eradicated. The study by Alvarez et al. [25] indicates that there has been a reemergence of the disease in dogs, with estimated prevalences of 18% in rural areas and 0.4% in peri-urban areas of the region.

For the mathematical modeling of CE transmission during the cycle of the disease in a rural area, work has been developed, mainly using compartmental models for dogs, sheep, and humans via ordinary differential equations [26–28]. In particular, as humans are involved in the tasks of deworming and culling dogs in rural areas, there is a risk that they may become accidentally infected. In the study by Wu et al. [26], the human is included in a model that initially only considered the transmission dynamics of the disease between dogs and sheep. On the other hand, recent investigations have identified increased mobility of the human population as a factor responsible for the emergence and re-emergence of infectious diseases, for example, Chagas disease and so-called airport ‘malaria’ [29–31]. The data-driven analysis through a correlation study by Khan et al. [30] justifies the spread of a global pandemic (H1N1) through international air travel. Using a mathematical model based on Markov chains and mobility data, Arenas et al. [31] found that the effect of human mobility on the spread of disease from one location to another is contingent upon population density and mobility patterns within those regions. A recent study by Gutiérrez-Jara et al. [32] examined the impact of human mobility on the spread of hantavirus. The findings suggest that human mobility plays a role in the incidence of this zoonotic disease.

In this study, we propose a mathematical model as a proof-of-concept to investigate the role of definitive and intermediate host mobility between peri-urban, urban and rural areas in the transmission and spread of echinococcosis caused by *Echinococcus granulosus* in its domestic cycle. The aim is to generate information that will improve our understanding of this zoonosis and facilitate the development of improvements in epidemiological surveillance, prevention and control processes. In particular, this host mobility factor may explain why it is difficult to maintain data from official sources that would allow us to know the real magnitude of the number of human cases affected by this health problem [1,14]. The mathematical modeling proposal is based on three classical compartmental epidemic models: S-E-I-R for humans, with a classification by age ranges; S-E-I for sheep; and S-I-S for dogs.

## Materials and methods

We constructed our model following recommendations outlined in the following sources: [26–28,32,33]. In particular, the core concepts of our mathematical model (excluding mobility) are based on the basic model of [26], which corresponds to a study of disease transmission in Qinghai province in China. Qinghai is an endemic livestock area with a prevalence between 7 and 10%. Given the epidemiological similarities between Qinghai Province in China and Chile, the numerical values of the initial conditions for the simulations were taken from [26], maintaining the order of magnitude. On the other hand, we constructed a range of numerical values for the parameters of the mathematical model of disease transmission dynamics based on data from several studies [26–28,34,35]. We assumed that the numerical values of the parameters associated with mobility.

We have included mobility in the mathematical modeling of CE because mobility affects the spread of infectious diseases. The role of host mobility between peri-urban, urban, and rural areas of an environment in the transmission and spread of canine and cystic echinococcosis caused by EG in its domestic cycle was analyzed using a compartmental mathematical model. Type S-E-I-R for age-structured accidental intermediate hosts (under 15 years and adults) that are susceptible, exposed (in the incubation period), infected, and removed (in our case deceased due to infection); Type S-E-I for usual intermediate hosts that are susceptible, exposed and infected; and Type S-I-S for definitive hosts that are susceptible and infected.

To address the role of host mobility in disease transmission and spread from both theoretical and general perspectives, we have employed a simplified strategic mathematical model. This approach allow us to gain insights into the dynamics of the phenomenon.

Our model is based on a system of deterministic ordinary differential equations (ODEs), that incorporate key epidemiological parameters of the infection along with host mobility factors.

**Fig 1:**
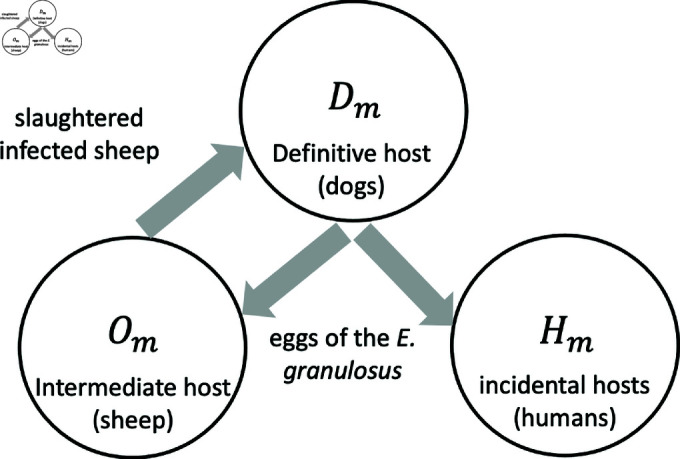
Visual model. Simplified visual model of the transmission dynamics of the disease between hosts.

We propose a mathematical model for the domestic life cycle of the disease (see Fig 1), involving the dog (*D*) as the definitive host and the sheep (*O*) as an intermediate host. When a human (*H*), child (*K*) or adult (*A*), is accidentally introduced into the transmission cycle of the disease, the average latency period before the manifestation of symptoms is approximately ten years following the initial infection.

The host populations were classified into the epidemiological states of susceptible (s), exposed (e), infected (i), and deceased from infection (d), as appropriate. Thus, by *X* we denote the size host population, with *X* ∈ { *D* , *O* , *K* , *A* } ,  by *m* the location zone and by *k* the epidemiological status, $N_{m}^{X}=\sum_{m,k}X_{m}^{k},$ with $N_{m}^{H}=N_{m}^{K}+N_{m}^{A}$, where $N_{m}^{X}$ corresponds to the size total host population *X* in zone *m* for each subpopulation in each of the *k* epidemiological statuses, respectively (see S1 Table).

**Fig 2:**
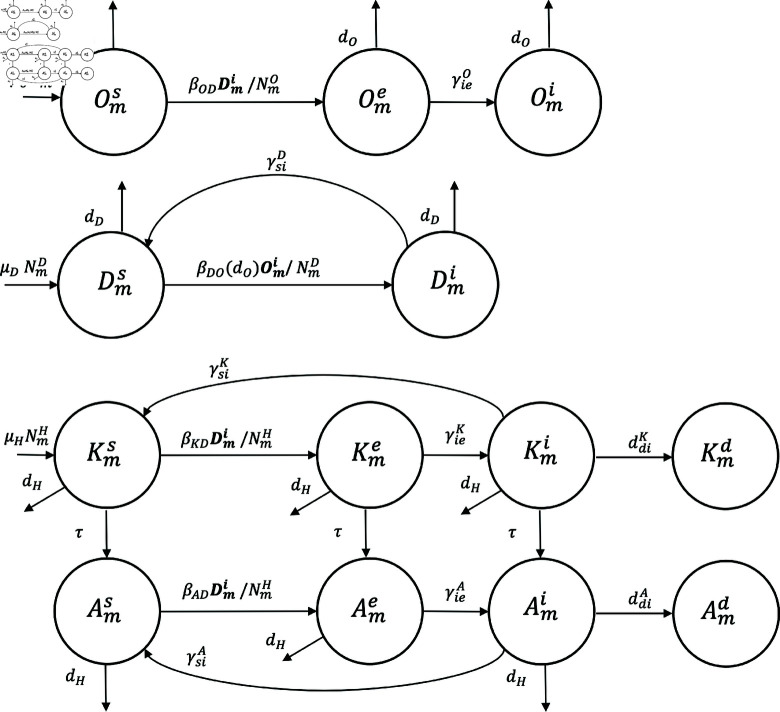
Compartmental diagram. Compartmental diagram for the transmission dynamics of cystic echinococcosis between dogs ($D_{m}$), sheep ($O_{m}$) and humans (children ($K_{m}$) and adults ($A_{m}$)) of the *m* area (peri-urban, urban and rural).

In our model, by $\mu_{X}$ and $d_{X}$ we denote the birth and natural mortality rate of host *X* ,  respectively; by $d_{di}^{H}$ we denote the mortality rate in humans resulting from infection; by $\beta_{DO}(d_{O})$ we denote the transmission rate of disease from sheep to dog is determined by the product of three factors. Firstly, the contact rate between the dog and infected viscera of dead sheep. Secondly, the probability of the dog becoming infected after such contact. And thirdly, the mortality rate of the sheep; Similarly, by $\beta_{KD},\beta_{AD}$ and $\beta_{OD}$ we denote the rates of disease transmission from dog to child, adult and sheep, respectively. It was assumed that the human remains exposed until the hydatid cyst causes clinical signs in an average time ($1∕\gamma_{ie}^{K}$ or $1∕\gamma_{ie}^{A}$). When this occurs, this host advances to the infected stage (see Fig 2). In this study, $1∕\gamma_{ie}^{O}$ is used to denote the half-life of the hydatid cyst in sheep.

**Fig 3:**
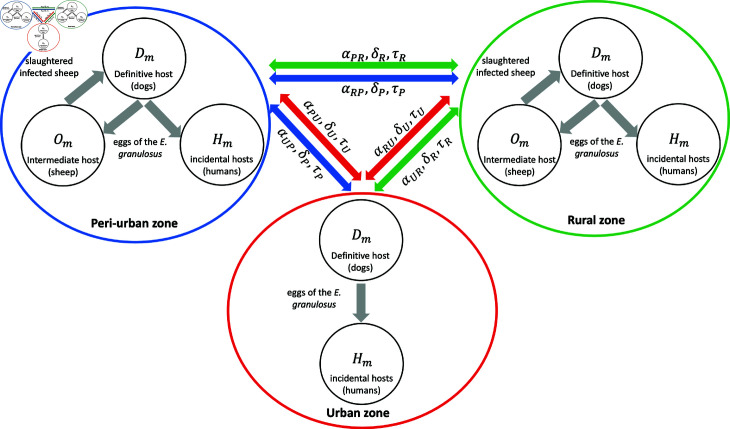
Visual model. Visual model of the transmission and spread dynamics of the disease between dogs ($D_{m}$), sheep ($O_{m}$) and humans ($H_{m}$) of *m* zones (peri-urban, urban and rural). *α* , *δ* and *τ* denote the host mobility parameters between zones.

We have divided the environment in which the disease is present into three zones: peri-urban (*P*), urban (*U*) and rural (*R*). S2 Table summarizes the parameters associated with host mobility between peri-urban, urban, and rural zones. $\delta_{a}^{X}$ is the rate at which host *X* outflow from zone *a* ,  $\alpha_{ab}^{X}$ is the fraction of hosts *X* from zone *b* that move to zone *a* ,  and $1∕\tau_{a}^{X}$ is the average time that a host *X* from zone *a* remains in another zone. For example, $\alpha_{PR}^{D}$ is the fraction of dogs from the rural area that move to the peri-urban area at a rate of $\delta_{R}^{D}$ and remains there an average time of $1∕\tau_{R}^{D}$ (see Fig 3).

This transmission scenario generally occurs in rural areas because dogs are frequently fed with animal viscera containing hydatid cysts, which initiates the parasite’s life cycle.

To incorporate the demographic factor in the host population into our modeling, we have assumed that the birth rate is proportional to the population size. Furthermore, we have assumed that $\mu_{X}=d_{X},$ which means that the total population size of dogs and sheep remains constant (see Fig 2). Finally, for simplicity, we have assumed that the residence time of human children in compartments $K_{m}^{k}$ with *m* ∈ { *P* , *U* , *R* }  and *k* ∈ { *s* , *e* , *i* }  has an exponential distribution, i.e., a larger fraction of these children have a short residence time in this compartment, while a smaller fraction of children leave after a longer time.

The graphs representing the mobility of host X between peri-urban, urban, and rural areas are shown in Fig 5. It should be noted that the modeling considered only the movements of the sheep from a rural to a peri-urban zone because this is what occurs in reality. Sheep is translated to the peri-urban area for subsequent home slaughter and human consumption. Fig 5 (a) shows that from zone *P* ,  a fraction $\alpha_{RP}$ of these hosts can move to zone *R* at a rate $\delta_{P}$ and remain there for an average time $1∕\tau_{P}.$ Another fraction, $\alpha_{UP},$ can move to zone *U* at a rate $\delta_{P}$ and remain there for an average time $1∕\tau_{P}.$ Similarly, these hosts can move from zone *U* to zones *P* and *R* ,  and from zone *R* to zones *P* and *U* .  Fig 5 (b) shows that from zone *R* ,  a fraction $\alpha_{PR}$ of these hosts can move to zone *P* at a rate $\delta_{R}$ and remain there for an average time $1∕\tau_{P}.$

**Fig 4:**
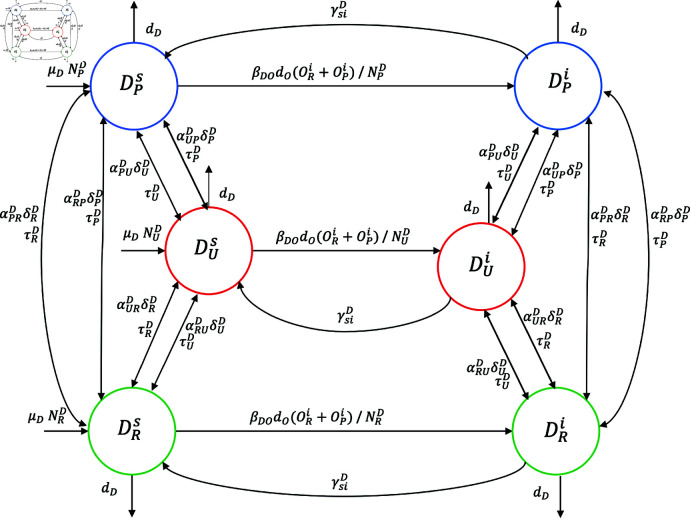
Graph for epidemiological dynamics and mobility. Graph for epidemiological dynamics and mobility in dogs between peri-urban (blue), urban (red), and rural (green) areas. The direction of the arrow indicates the direction of host movement.

**Fig 5:**
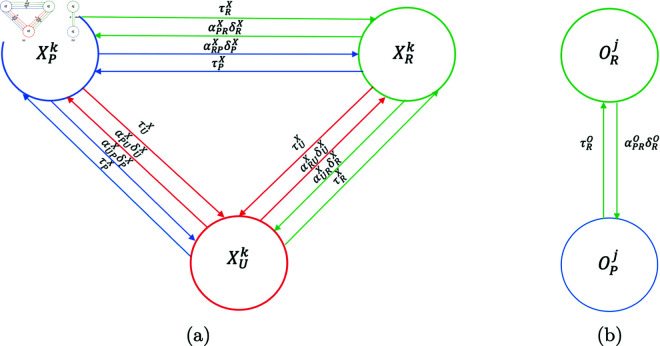
Graph of host mobility between peri-urban, urban , and rural areas. Graph of host mobility between peri-urban (blue), urban (red), and rural (green) areas; (a) host *X* (dog or human) and (b) host *O* (sheep). The direction of the arrow indicates the direction of host movement. The *α* fraction of the host moves to the other two zones at a rate of *δ*, where it remains for an average time of 1 ∕ *τ* .

**Fig 6:**
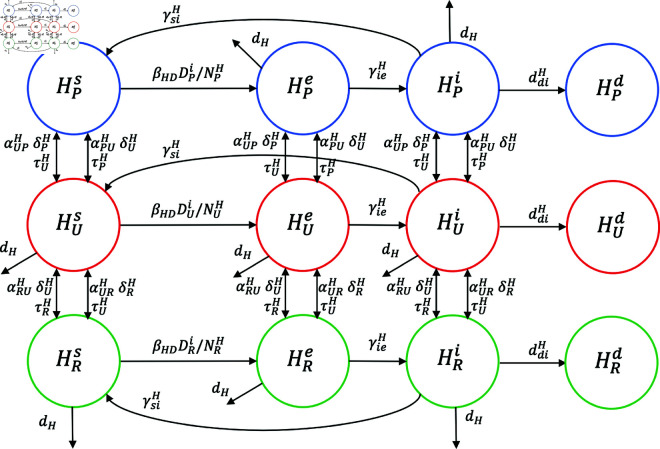
Graph for epidemiological dynamics and mobility in humans. Graph for epidemiological dynamics and mobility in humans (children and adults) between peri-urban (blue), urban (red), and rural (green) areas. The direction of the arrow indicates the direction of host movement.

**Fig 7:**
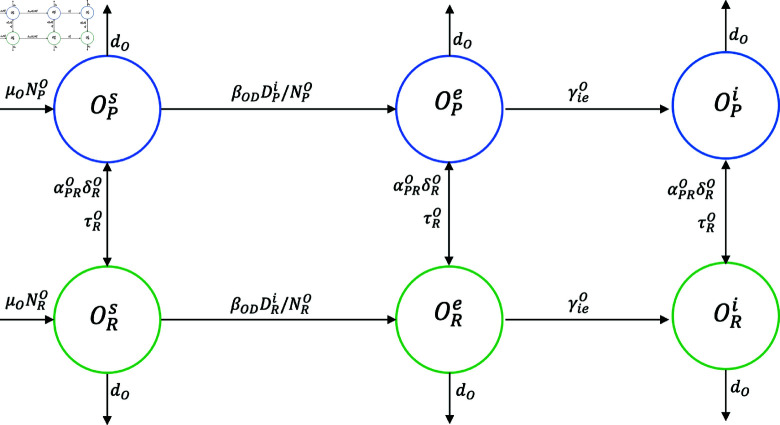
Graph for epidemiological dynamics and mobility in the sheep. Graph for epidemiological dynamics and mobility in the sheep between peri-urban (blue) and rural (green) areas. The direction of the arrow indicates the direction of the host movement.

The dynamics of transmission and spread of the disease are represented in Figs 4–7, which show the epidemiological stages and host mobility. Fig 4 shows that a dog in the urban area (*U*) can enter the $D_{U}^{s}$ state at rate $\mu_{D}$ and remain there until its natural death at a rate $d_{D}$ or it has the following two alternatives: to become infected at rate $\beta_{DO}d_{O}$ and enter state $D_{U}^{i},$ participate in the proportion $\alpha_{PU}^{D}$ of urban dogs moving to a peri-urban area at rate $\delta_{U}^{D}$ and stay in the other zone (*P* , *R*) for an average amount of time $1∕\tau_{U}^{D}$.

Analogously, for the human host, in Fig 6, a susceptible child in the peri-urban area, belonging to the $K_{P}^{s},$ state, may remain in that epidemiological stage until adulthood, $A_{P}^{s},$ at rate *τ* and then become infected by direct contact with an infected dog at rate $\beta_{AD}$ and enter the $A_{P}^{e}$ state.

In Fig 7 it is possible to observe that a sheep in the rural area (*R*) can enter the $O_{R}^{s}$ state at rate $\mu_{O}$ and remain there until its natural death at a rate $d_{O}$ or: become infected by consuming food or water contaminated with dog feces containing viable EG eggs at rate $\beta_{OD}$ or participate in the $\alpha_{PR}^{O}$ proportion of rural sheep moving into a peri-urban area at rate $\delta_{R}^{O}$ and remain there until slaughter and subsequent human consumption.

The graphs in Figs 4–7 describing the transmission and spread dynamics of canine and cystic echinococcosis, which were proposed in this work, can be represented mathematically by the system of ordinary differential equations:


$$\begin{array}{cccc} {Ẋ}_{P} & =-(\Gamma_{UP}+\Gamma_{RP})X_{P}+\Gamma_{PU}X_{U}+\Gamma_{PR}X_{R}+\Omega_{P}X_{P} & & \\ {Ẋ}_{U} & =\Gamma_{UP}X_{P}-(\Gamma_{RU}+\Gamma_{PU})X_{U}+\Gamma_{UR}X_{R}+\Omega_{U}X_{U} & & \\ {Ẋ}_{R} & =\Gamma_{RP}X_{P}+\Gamma_{RU}X_{U}-(\Gamma_{PR}+\Gamma_{UR})X_{R}+\Omega_{R}X_{R} & \end{array}$$ (1)

Where; $\Omega_{\omega }$ is the epidemiological parameter of the host *X* in area *w* ,  $\Gamma_{ba}:=\alpha_{ba}^{x_{a}}\delta_{a}^{x_{a}}$  +  $\tau_{a}^{x_{b}}$ is the mobility parameter associated with the movement of a population of hosts from area *a* to *b* ,  whichever they are, composed of the proportion of hosts from area $a,x_{a},$ moving to area *b* at rate $\delta_{a}^{x_{a}}$ and ${\left(\tau_{a}^{x_{b}}\right)}^{-1}$ the average time spent in area *b* by hosts from area $a,x_{b};$ $X_{P}=[D_{P}^{k_{1}},O_{P}^{k_{2}},K_{P}^{k_{3}},A_{P}^{k_{3}}]\in {\mathbb{R}}^{13},$ $X_{U}=[D_{U}^{k_{1}},K_{U}^{k_{3}},A_{U}^{k_{3}}]\in {\mathbb{R}}^{10},$ $X_{R}=[D_{R}^{k_{1}},O_{R}^{k_{2}},K_{R}^{k_{3}},A_{R}^{k_{3}}]\in {\mathbb{R}}^{13},$ $k_{1}\in \{s,i\},k_{2}\in \{s,e,i\},k_{3}\in \{s,e,i,d\}.$ The extended version of system (1) can be found in S1 Appendix S1 Appendix.

To qualitatively analyze the role that host mobility plays in the transmission and spread of echinococcosis between peri-urban, urban, and rural areas of an environment, the system (1) was solved numerically using a computational code that utilized the internal function ode45 (based on a Runge-Kutta type numerical method) of MATLAB version R2022a [36]. For the numerical simulations, the values recorded in S2 Table and S3 Table (see S2 and S3 Tables) for the parameters and initial data, respectively, were considered. To illustrate the phenomenon of adult cases inadvertently infected during childhood due to undetected disease, we simulated their dynamics over a 20-year period.

The basic reproductive number, $R_{0},$ is a theoretical parameter in epidemiology that provides insight into the impact of an infectious disease in the early stages of its spread and is defined as “the average number of secondary cases that an infectious individual will produce in a population consisting only of susceptible individuals" [37]. Mathematically, $R_{0}$ is closely related to the parameters used to model the transmission and spread of any infectious disease. To calculate the reproductive number, the following generation matrix [38,39] is associated with the system of differential equations. Since the expression obtained for $R_{0}$ will depend on the model parameters, different disease scenarios were established to demonstrate the impact of host mobility on transmission and spread. Therefore, the following scenarios were chosen: without host mobility ($R_{0}^{*}$), with partial host mobility in each zone of an environment ($R_{0P}$ and $R_{0R}$) and with total host mobility in one environment ($R_{0}$). Where the following expressions are obtained:


$$\begin{array}{llll} R_{0}^{*} & =(\frac{\beta_{DO}\gamma_{ie}^{O}}{\left(\gamma_{ie}^{O}+d_{O}\right)}{)}^{1∕2}(\frac{\beta_{OD}}{\left(\gamma_{si}^{D}+d_{D}\right)}{)}^{1∕2};\;R_{0P}={\left(\chi_{1}\right)}^{1∕2}{\left(\chi_{2}\right)}^{1∕2};\;R_{0R}={\left(\chi_{3}\right)}^{1∕2}{\left(\chi_{4}\right)}^{1∕2};\; & & \;\\ \chi_{1} & =\frac{\left(\beta_{OD}∕N_{P}^{O})(\mu_{O}N_{P}^{O}+\alpha_{PR}^{O}\delta_{R}^{O}O_{R}^{s*}\right)}{\left(2\tau_{P}^{D}+\delta_{P}^{D}+d_{D}+\gamma_{si}^{D})(\tau_{P}^{O}+d_{O}\right)},\; & & \;\\ \chi_{2} & =\frac{\left(\beta_{DO}d_{O}∕N_{P}^{D})\gamma_{ie}^{O}(\mu_{D}N_{P}^{D}+(\alpha_{PR}^{D}\delta_{R}^{D}+\tau_{R}^{D})D_{R}^{s*}+(\alpha_{PU}^{D}\delta_{U}^{D}+\tau_{U}^{D})D_{U}^{s*}\right)}{\left(\tau_{P}^{O}+\gamma_{ie}^{O}+d_{O})(2\tau_{P}^{D}+\delta_{P}^{D}+d_{D})(\tau_{P}^{O}+d_{O}\right)},\; & & \;\\ \chi_{3} & =\frac{\left(\beta_{DO}d_{O}∕N_{R}^{D})\gamma_{ie}^{O}(\mu_{D}N_{R}^{D}+(\alpha_{RP}^{D}\delta_{P}^{D}+\tau_{P}^{D})D_{P}^{s*}+(\alpha_{RU}^{D}\delta_{U}^{D}+\tau_{U}^{D})D_{U}^{s*}\right)}{\left(\alpha_{PR}^{O}\delta_{R}^{O}+\gamma_{ie}^{O}+d_{O})(2\tau_{R}^{D}+\delta_{R}^{D}+d_{D})(\alpha_{PR}^{O}\delta_{R}^{O}+d_{O}\right)},\; & & \;\\ \chi_{4} & =\frac{\left(\beta_{OD}∕N_{R}^{O})(\mu_{O}N_{R}^{O}+\tau_{P}^{O}O_{P}^{s*}\right)}{\left(d_{D}+\gamma_{si}^{D}+2\tau_{R}^{D}+\delta_{R}^{D})(\alpha_{PR}^{O}\delta_{R}^{O}+d_{O}\right)},\; & & \;\\ \; & \; & \end{array}$$

and


$$\begin{array}{lll} R_{0}=\frac{{\left(AB\right)}^{1∕2}}{C}. & \; & \end{array}$$

Where, as in $R_{0P}$ and $R_{0R},R_{0}$ also shows the dependence on the mobility parameters (see *A* , *B* ,  and *C* defined in S2 Appendix S2 Appendix).

In addition, to determine the impact that a change of a certain parameter can have on the value of $R_{0},$ a sensitivity analysis on $R_{0}$ with respect to the parameter was developed using the definition given in [37]:


$$\begin{array}{ccc} {𝜖}_{p}^{R_{0}}=\frac{\partial R_{0}}{\partial p}\frac{p}{R_{0}}\approx \frac{\%△R_{0}}{\%△p} & & \end{array}$$ (2)

The numerical value of the sensitivity on $R_{0}$ for *p* can be positive or negative. When ${𝜖}_{p}^{R_{0}}>0,$ an increase in the numerical value of the parameter *p* leads to an increase in $R_{0};$ when it is negative the opposite happens. Approximately, the expression given in (2) says that 1% change in the numerical value of the parameter *p* leads to ${𝜖}_{p}^{R_{0}}\%$ change in $R_{0}.$

## Results

This section shows the results of the numerical solutions of the mathematical model proposed in this work for disease dynamics with and without host mobility between peri-urban, urban, and rural areas of an environment.

### Impact of mobility on the number of infected

**Fig 8:**
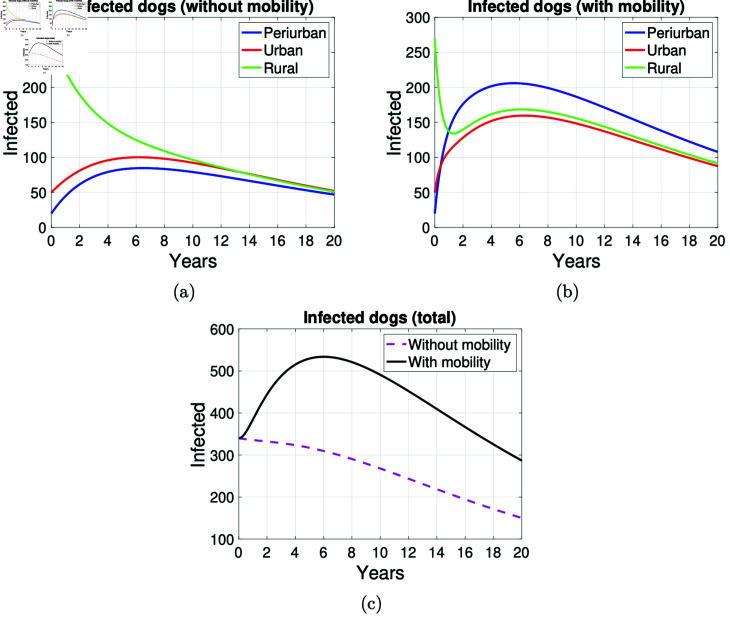
Graphs on the mobility of infected dogs. Graphs on the mobility of infected dogs in the areas *P* , *U* and *R* .  Simulation made at 20 years. (a) Infected dogs without mobility, (b) infected dogs with mobility in the three areas, and (c) total infected dogs with and without mobility.

Using the methodology described in the previous section, Fig 8 presents graphs of dog infection dynamics over a 20-year simulation period. The total population size of dogs in the peri-urban, urban and rural areas considered for the simulations was 520, 4050 and 1770, respectively. The initial population of infected dogs in these areas was 20, 50, and 270, respectively. We choose the proportions of initially infected hosts in each zone for illustrative purposes only (see S3 Table). However, as reported in [25], the highest prevalence of EG is found in rural areas. Consequently, we have initially considered that the highest proportion of infected dogs and sheep is found in these areas. In each zone, the dynamics of infection transmission are shown.

The simulation shown in Fig 8(a) assumes that dogs do not move between the different zones and are infected at the same rate of disease transmission from sheep to dog in the three zones. Specifically, Fig 8(a) presents the behavior of infected dog populations during 20 years of numerical simulation. In general terms, the graphs show the behavior of canine echinococcosis dog populations in the peri-urban (blue curve), urban (red curve), and rural (green curve) zones of an environment over that period without assuming the mobility of dogs between zones. The number of infected dogs in the peri-urban and urban zones grows and peaks around year 6 of the simulation and then slowly decreases. In the rural zone, the number of infected dogs decreases by approximately half by year 6. In all zones, the number of infected dogs decreases at a similar rate from year ten until each zone has roughly the same number of infected dogs (50).

The simulation results assuming mobility of dogs between peri-urban, urban, and rural areas of an environment are shown in Fig 8(b). Here, the number of infected dogs in the rural zone decreases rapidly until year one and then begins to grow as fast as the number of infected dogs in the urban zone, reaching the maximum number of infected dogs in year 6. The number of infected dogs in the peri-urban zone grows more rapidly until about year 2, after which the number of infected dogs grows and decreases at the same rate in all three zones until, on average, 100 infected dogs at the end of the simulation.

The curves in Fig 8(c) show that the total number of infected dogs increases when mobility of dogs between areas of an environment is assumed. The number of infected dogs increases, accelerates until it peaks in year six, and then begins to decline until it ends up with approximately twice as many infected dogs as if no mobility were assumed.

**Fig 9:**
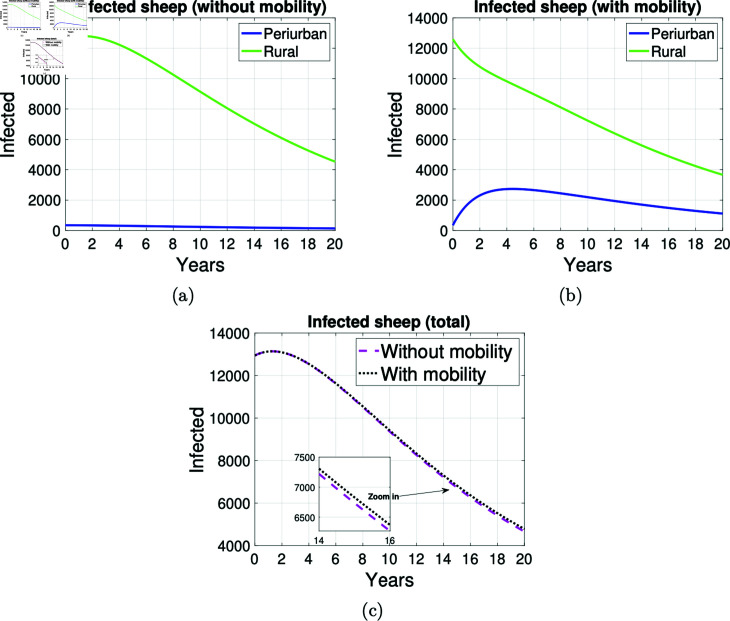
Graphs on the mobility of infected sheep. Graphs on the mobility of infected sheep in the areas *P* and *R* .  Simulation made at 20 years. (a) Infected sheep without mobility, (b) infected sheep with mobility in both areas and (c) total infected sheep with and without the possibility of mobility.

The numerical simulation of the dynamics of cystic echinococcosis transmission to sheep through consumption of food contaminated with canine echinococcosis-infected dog feces in the peri-urban and rural areas of an environment is shown in Fig 9. In general terms, when comparing Fig 9a and 9b, here, the number of infected sheep in the rural area decreases less rapidly when mobility is assumed. However, the number of infected sheep in the peri-urban zone increases until it reaches its maximum in the fourth year. It slowly decreases until the end of the simulation when there are about 1000 infected sheep in this zone.

Figs 8(b) and 9(b) show that the mobility of infected sheep from rural to peri-urban areas has a direct impact on the increase in the number of infected dogs in the peri-urban area.

**Fig 10:**
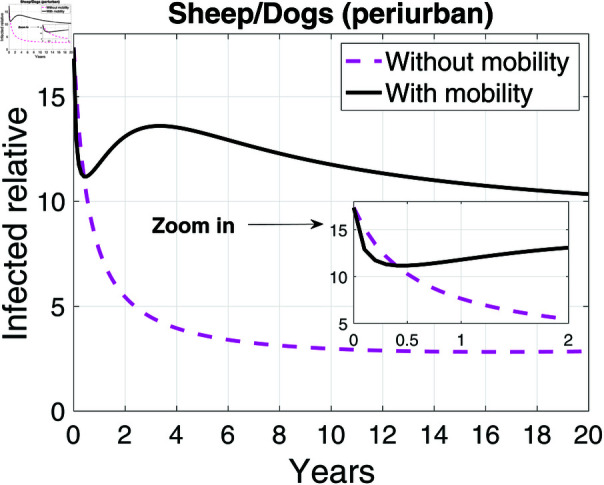
Infected sheep per infected dogs. Graph showing the number of infected sheep per infected dogs in the peri-urban area with and without the possibility of mobility and a sub-graph (zoomed in) of the Infected relative in the first two years of the simulation.

Incorporating host mobility into the model resulted in a notable increase in the proportion of infected sheep compared to infected dogs, beginning around the first year (see Fig 10). This proportion remained above 10 in the model that incorporated host mobility, while in the model that did not take it into account, it was below 5 from the second year onwards. This comparative analysis underscores the significant impact of mobility on the transmission dynamics of the infection. Our results indicate that in the presence of mobility, for every infected dog, there are approximately twice as many infected sheep as compared to the scenario without mobility.

**Fig 11:**
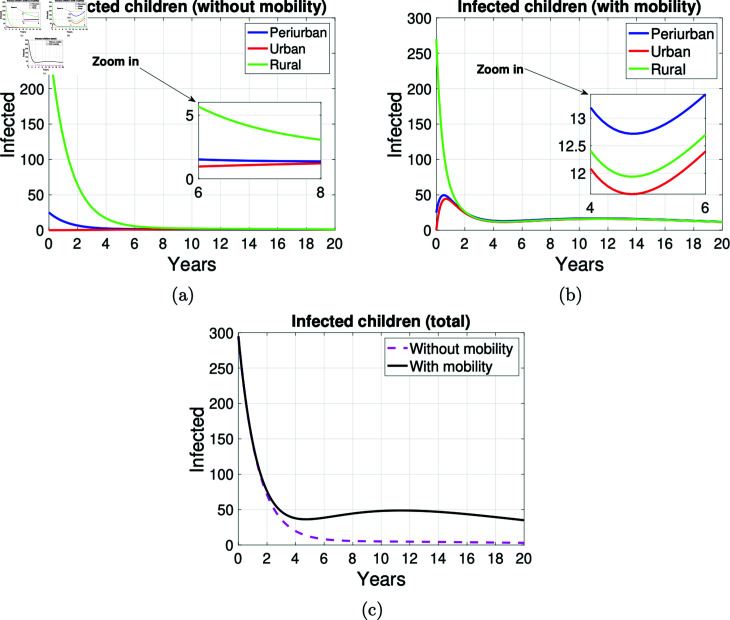
Graphs on the mobility of infected children. Graphs on the mobility of infected children in the areas *P* , *U* and *R* .  Simulation made at 20 years. (a) Infected children without the possibility of mobility and a sub-graph (zoomed in) of the infected between six and eight years of the simulation is presented, (b) infected children with mobility in the three areas and a sub-graph (zoomed in) of the infected between four and six years of the simulation is presented, and (c) total infected children with and without the possibility of mobility.

**Fig 12:**
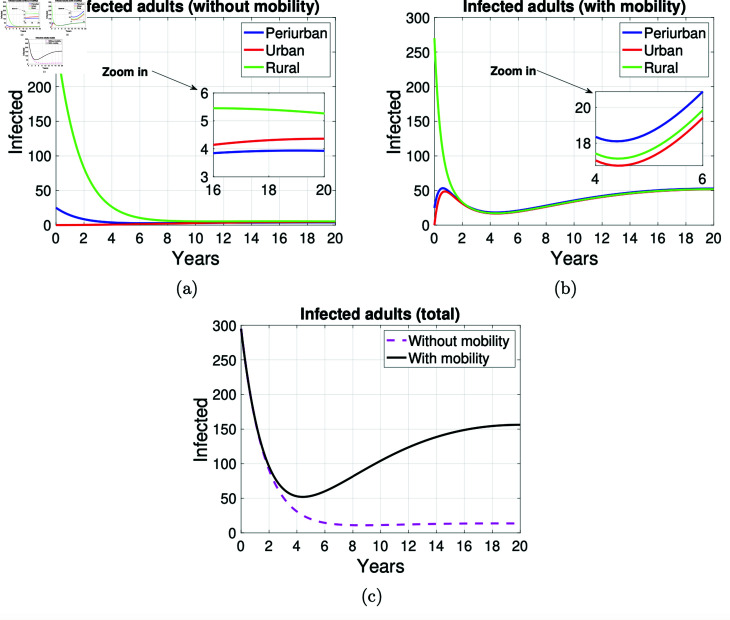
Graphs on the mobility of infected adults. Graphs on the mobility of infected adults in the areas *P* , *U* and *R* .  Simulation made at 20 years. (a) Infected adults without mobility and a sub-graph (zoomed in) of the infected between sixteen and twenty years of the simulation is presented, (b) infected adults with mobility in the three areas and a sub-graph (zoomed in) of the infected between four and six years of the simulation is presented, and (c) total infected adults with and without mobility.

In rural areas, the number of human cases (children and adults) decreases faster than in peri-urban areas, as shown in Figs 11(a) and 12(a). Considering that human cases occur through consumption of contaminated food or through contact with infected dogs, these figures show that the disease is maintained from childhood to adulthood and does not disappear after 20 years of simulation. In particular, the far right of the graphs in Fig 12(a) shows that human cases of adult are maintained. In the graphs in Figs 11(b) and 12(b), the mobility of children and adults in the three zones is always associated with an increase in the number of cases. According to the simulation results, as can be seen from the graphs in Figs 11(b) and 11(c) and 12(b) and 12(c), the mobility of people has an impact on the spread of disease between peri-urban, urban and rural areas of an environment, from the graphs in Figs 11(c) and 12(c) appears that independently of mobility, the number of human cases decreases at the same rate until approximately the first year. However, from year four onwards, the number of adult cases grows until the end of the simulation when mobility is assumed and remains constant when mobility is not considered.

**Fig 13:**
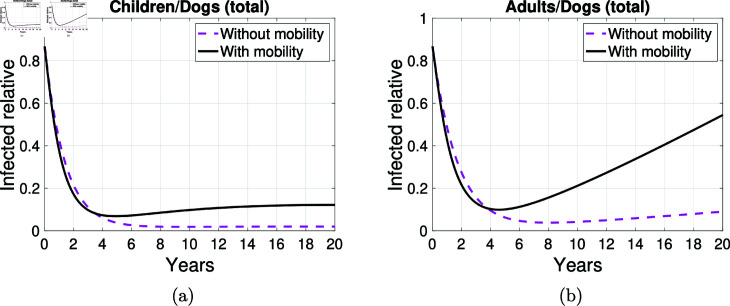
Infected humans per infected dogs. Graph showing the number of infected humans per infected dogs with and without the possibility of mobility between the three areas (total).

Fig 13 illustrates that mobility is responsible for an increase in the proportion of human cases compared to dog cases from approximately the fourth year onwards. Furthermore, it can be observed that the growth of this proportion is more pronounced when considering adult humans. However, over the 20-year period, this proportion remains below 0.6, indicating that for every 10 infected dogs, there are at most 6 adult human cases. In particular, in year 5, Fig 13(a) shows that in the absence of mobility, there is approximately one child with echinococcosis for every 30 infected dogs. This figure increases to two when mobility is assumed. Similarly, Fig 13(b) illustrates that, in the absence of mobility, there are approximately two adult cases for every 30 infected dogs. This figure increases to three when mobility is assumed.

### Sensitivity on $R_{0}$

Numerical simulations of the mathematical model proposed as a tool for understanding the mechanism of transmission and spread of echinococcosis are subject to the precise ignorance of the numerical values of the parameters involved [40,41]. In particular, this infectious disease has been neglected, and in the field of mathematical modeling, there is a lack of research regarding the parameters (transfer rates between compartments) [34]. For example, in the studies we have reviewed so far, there has been no consideration of time-varying rates of disease transmission. The robustness of simulations and the role of the parameters is quantified using the sensitivity on $R_{0}$ with respect to each of the parameters of the proposed model [37]. Let


$$\begin{array}{ccc} {𝜖}_{p}^{R_{0}}=\frac{\partial R_{0}}{\partial p}\frac{p}{R_{0}}\approx \frac{\%△R_{0}}{\%△p} & & \end{array}$$ (3)

the sensitivity index on $R_{0}$ with respect to the parameter *p* ,  where; $p\in \{\beta_{OD},\beta_{DO},\gamma_{si}^{D},\gamma_{ie}^{O},d_{D},$ $d_{O},\delta_{P}^{D},\delta_{U}^{D},\delta_{R}^{D},$ $\tau_{P}^{D},\tau_{U}^{D},$ $\tau_{R}^{D},\alpha_{PU}^{D},$ $\alpha_{UP}^{D},\alpha_{RP}^{D},$ $\alpha_{PR}^{D},\alpha_{UR}^{D},\alpha_{RU}^{D}\}.$

**Fig 14:**
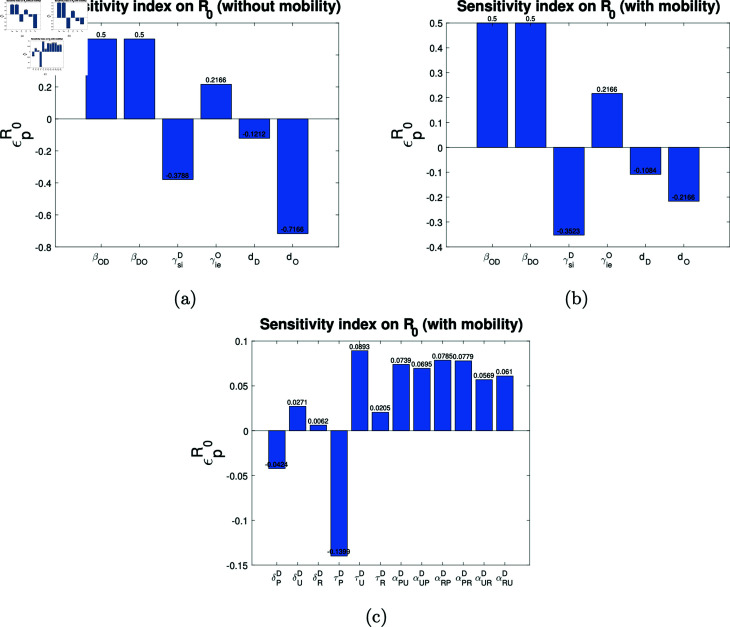
The sensitivity index on $R_{0}.$ Graphical representation of the sensitivity index on $R_{0}$ with respect to a; (a) epidemiological parameters (without mobility), (b) epidemiological parameters (with mobility) and (c) mobility parameters.

From the Fig 14, it can be seen that the impact of alterations to parameters $\beta_{OD},\beta_{DO},\gamma_{si}^{D},\gamma_{ie}^{O}$ and $d_{D}$ on $R_{0}$ is similar across both models (with and without mobility). However, an increase in the sheep mortality rate ($d_{O}$) has a more pronounced effect on the decline in $R_{0}$ when mobility is not taken into account. Specifically, when $d_{O}$ is increased by 1%, the decrease in $R_{0}$ is 0.2166% and 0.7166% in models with and without mobility, respectively. Furthermore, as illustrated in Fig 14b and 14c, the magnitudes that reach the sensitivity index on $R_{0}$ with respect to the epidemiological parameters are greater than those of the mobility parameters. This indicates that greater changes in $R_{0}$ can be achieved with changes in the epidemiological parameters than with changes in the mobility parameters.

## Discussion

As a result of field and laboratory work carried out at a particular time according to a descriptive approach based on data collected in studies [17,20,22,25], recommendations for the control of echinococcosis are given. However, the transmission mechanics of this disease in hosts are not explicitly considered. Our proposal as a proof-of-concept, based on mathematical modeling, is more general because it allows the analysis of different scenarios by modifying the numerical values of the parameters or the initial conditions of the other state variables according to the epidemiological stages of each host.

The numerical solutions of the proposed mathematical model, obtained in the simulations, suggest that the mobility of dogs and sheep increases the number of cases of echinococcosis and, therefore, the disease has a greater spread and, as noted in [17], in the absence of adequate control, there is the possibility of increased transmission to humans. Thus, future work could include, in the mathematical modeling, parameters associated with control measures, such as deworming of dogs and vaccination of sheep, to evaluate their impact on the prevalence of the disease in humans as they move between areas. Additionally, parameters affecting the compartments describing the epidemiologic stages of cystic echinococcosis in children and adults can be included, based on the measures proposed in [42] in relation to early treatment of cases and improved determination of the prevalence of infection in the human population.

Initially, our simulations show a prevalence of 15.48% in rural areas. However, as reported in [14], human cases are underreported, i.e., there is a gap between the number of registered and unregistered cases. This fact, the existence of a higher real prevalence of the disease, could be explained by our simulations that include the factor of host mobility between peri-urban, urban and rural areas because when this factor is not considered the prevalence is lower in all areas.

As is known, in mathematical terms, the basic reproductive number, $R_{0},$ is closely related to the parameters of the mathematical model used to describe the dynamics of the transmission and spread of any infectious disease. The sensitivity on $R_{0}$ with respect to a model parameter is an indicator that allows us to determine the effect on $R_{0}$ when there is a change in the numerical value of that parameter. In our work, we found that approximately a 1% increase in the rate of transmission of infection among non-human hosts results in a 0.5% increase in the basic reproductive number of the disease; the same result is reported in the works of Chacha et al. [34], Hartung et al. [28] and Getachew et al. et al. [35]. In agreement with our results, the studies by authors [28], [34], and [35] revealed that an increase in the parameter associated with the incubation period of infection in sheep ($\gamma_{ie}^{O}$) also increases the value of the basic reproductive number for the proposed mathematical model. Other parameters that change the numerical value of $R_{0}$ ($|{𝜖}_{p}^{R_{0}}|=0.5$) wich were included in the works of Hartung et al. [28] and Getachew et al. [35] but not considered in this work are the rate of contamination with eggs at the environment by infected dogs and the concentration of EG eggs in which half of all contacts with sheep produce infection.

From Fig 14(b), it can be seen that an increase in the numerical value of the parameters $\gamma_{ie}^{O}$ and $d_{O}$ has an effect of the same magnitude on $R_{0}.$ However, in one case $R_{0}$ increases and in another case $R_{0}$ decreases. That is, a 1% increase in $\gamma_{ie}^{O}$ (with the other parameters held fixed) increases the value of $R_{0}$ by 0.2166%, and a 1% increase in $d_{O}$ (with the other parameters held fixed) decreases the value of $R_{0}$ by 0.2166%. This suggests that shortening the incubation period in the sheep will increase $R_{0}$ and increasing the natural mortality rate of the sheep will decrease $R_{0}.$

In our work, we interpret $R_{0}$ as an indicator of the initial transmission and spread of echinococcosis in an environment composed of peri-urban, urban, and rural areas. This indicator is typically contingent upon epidemiological parameters and mobility patterns exclusively associated with the hosts that transmit the disease. Gutiérrez-Jara et al. [32] investigated the spread of hantavirus and identified that epidemiological parameters and human mobility influence its transmission. Thus, the biological mechanism underlying the transmission of the disease should be considered in the mathematical expression of a quantitative indicator of the transmission of an infectious disease.

Chile has a total length of 4,300 km and is divided into fifteen administrative regions. At present, the country lacks an official national epidemiological surveillance program for the detection of EG in dog feces or sheep infection. Nevertheless, significant research has been conducted, including the study by Álvarez et al. [25], which sampled dog feces in epidemiological units (EU) in the Magallanes region, where the parasite is endemic. Of the total number of samples tested, 18% in rural areas and 0.4% in peri-urban areas were found to be positive for the parasite. In a separate study by Álvarez et al. [42], a high prevalence of the disease was documented in dogs in rural and urban areas within the Coquimbo region, with rates oscillating between 3.5% and 11.7%. The national incidence of CE in sheep is 31 cases per 1,000, with the most affected regions being Los Lagos, Araucanía, Los Ríos, Aysén, and Magallanes. At present, the epidemiological situation in humans is characterized by high, medium and low endemic scenarios, and there is currently no national program in place for the control of the disease [4,8]. Sensitivity indexes on $R_{0},$ which consider the fraction of the dog population that moves between regions, indicate that an increase in this fraction leads to an increase in $R_{0}.$ These results, together with the knowledge of the areas affected by the disease, are fundamental to direct efforts to control dog movements and, thus, prevent the transmission of the disease to humans and sheep.

Our research on how mobility impacts the transmission and spread of cystic echinococcosis has the potential to influence future strategies significantly. It supports choosing control measures that fit the regional reality, making it a crucial study area. Despite the limited data on the disease in Chile, our strategic model is designed to understand the local realities where the mobility of specific hosts impacts the infection in humans. According to our model and study, the transmission and spread of EG on dogs and sheep with high mobility maintain a high prevalence in zones such as Magallanes. Future work can be oriented to generate designs or strategies optimized, i.e., focused on key actors, for data collection to overcome data limited and outdated and to strengthen the mathematical modeling and its interpretations.

## Conclusion

The distance between the curves of total infected human hosts, with and without mobility, suggests that the inclusion of the mobility factor captures its influence on both reported human cases and hospital discharges of cystic echinococcosis. The parameters included in the mathematical expression of $R_{0}$, a key indicator of disease transmission, highlight the importance of targeting both definitive and intermediate hosts for effective control measures. In addition, the sensitivity analysis on $R_{0}$ indicates the need for greater precision in determining which parameters have the greatest impact ($|{𝜖}_{p}^{R_{0}}|\geq 0.1399$) on $R_{0},$ namely: the rates of disease transmission between non-human animal hosts, the incubation period of infection in the sheep, the infectious period of the dog, the mortality rate of the sheep, and the length of time the infected dog remains in the peri-urban area. Our mathematical model, as a proof-of-concept, is a valuable tool for understanding the impact of host mobility on the transmission and spread of canine and cystic echinococcosis.

## Supporting information

S1 Appendix Proposed mathematical model. (PDF)

S2 Appendix$R_{0}$ for proposed mathematical model.(PDF)

S1 Table State variable definitions. (PNG)

S2 TableDefinitions and values of mobility and epidemiological parameters (unit: $yr^{-1}$).(PNG)

S3 Table Initial host values according to epidemiological stages and area. (PNG)
