# Supplementary material for: The role of host mobility in the transmission and spread of Echinococcus granulosus: A Chile-based mathematical modeling approach
Source: PLoS Negl Trop Dis. 2025 Apr 14;19(4):e0012948. doi: 10.1371/journal.pntd.0012948 (PMC11996221; doi:10.1371/journal.pntd.0012948)
Supplement: S1 Appendix — (PDF) [file pntd.0012948.s001.pdf]

S1 Appendix

Proposed mathematical model

$$
\begin{aligned}
\dot{D}^s_P &= (\alpha^D_{PR}\delta^D_R + \tau^D_R)D^s_R + (\alpha^D_{PU}\delta^D_U + \tau^D_U)D^s_U - (2\tau^D_P + \delta^D_P)D^s_P \\
&\quad + \mu_D N^D_P + \gamma^D_{si}D^i_P - \Big(\frac{\beta_{DO}d_O(O^i_P + O^i_R)}{N^D_P} + d_D\Big)D^s_P \\
\dot{D}^i_P &= (\alpha^D_{PR}\delta^D_R + \tau^D_R)D^i_R + (\alpha^D_{PU}\delta^D_U + \tau^D_U)D^i_U - (2\tau^D_P + \delta^D_P)D^i_P \\
&\quad + \frac{\beta_{DO}d_O(O^i_P + O^i_R)}{N^D_P}D^s_P - (d_D + \gamma^D_{si})D^i_P \\
\dot{D}^s_U &= (\alpha^D_{UP}\delta^D_P + \tau^D_P)D^s_P + (\alpha^D_{UR}\delta^D_R + \tau^D_R)D^s_R - (2\tau^D_U + \delta^D_U)D^s_U \\
&\quad + \mu_D N^D_U + \gamma^D_{si}D^i_U - \Big(\frac{\beta_{DO}d_O(O^i_P + O^i_R)}{N^D_U} + d_D\Big)D^s_U \\
\dot{D}^i_U &= (\alpha^D_{UP}\delta^D_P + \tau^D_P)D^i_P + (\alpha^D_{UR}\delta^D_R + \tau^D_R)D^i_R - (2\tau^D_U + \delta^D_U)D^i_U \\
&\quad + \frac{\beta_{DO}d_O(O^i_P + O^i_R)}{N^D_U}D^s_U - (d_D + \gamma^D_{si})D^i_U \\
\dot{D}^s_R &= (\alpha^D_{RP}\delta^D_P + \tau^D_P)D^s_P + (\alpha^D_{RU}\delta^D_U + \tau^D_U)D^s_U - (2\tau^D_R + \delta^D_R)D^s_R \\
&\quad + \mu_D N^D_R + \gamma^D_{si}D^i_R - \Big(\frac{\beta_{DO}d_O(O^i_P + O^i_R)}{N^D_R} + d_D\Big)D^s_R \\
\dot{D}^i_R &= (\alpha^D_{RP}\delta^D_P + \tau^D_P)D^i_P + (\alpha^D_{RU}\delta^D_U + \tau^D_U)D^i_U - (2\tau^D_R + \delta^D_R)D^i_R \\
&\quad + \frac{\beta_{DO}d_O(O^i_P + O^i_R)}{N^D_R}D^s_R - (d_D + \gamma^D_{si})D^i_R \\
\dot{O}^s_P &= \alpha^O_{PR}\delta^O_R O^s_R - \tau^O_P O^s_P + \mu_O N^O_P - \Big(\frac{\beta_{OD}D^i_P}{N^O_P} + d_O\Big)O^s_P \\
\dot{O}^e_P &= \alpha^O_{PR}\delta^O_R O^e_R - \tau^O_P O^e_P + \frac{\beta_{OD}D^i_P O^s_P}{N^O_P} - \Big(\gamma^O_{ie} + d_O\Big)O^e_P \\
\dot{O}^i_P &= \alpha^O_{PR}\delta^O_R O^i_R - \tau^O_P O^i_P + \gamma^O_{ie}O^e_P - d_O O^i_P \\
\dot{O}^s_R &= \tau^O_P O^s_P - \alpha^O_{PR}\delta^O_R O^s_R + \mu_O N^O_R - \Big(\frac{\beta_{OD}D^i_R}{N^O_R} + d_O\Big)O^s_R \\
\dot{O}^e_R &= \tau^O_P O^e_P - \alpha^O_{PR}\delta^O_R O^e_R + \frac{\beta_{OD}D^i_R O^s_R}{N^O_R} - \Big(\gamma^O_{ie} + d_O\Big)O^e_R \\
\dot{O}^i_R &= \tau^O_P O^i_P - \alpha^O_{PR}\delta^O_R O^i_R + \gamma^O_{ie}O^e_R - d_O O^i_R
\end{aligned}
$$

$$
\begin{aligned}
\dot{K}_P^s &= (\alpha_{PR}^K \delta_R^K + \tau_R^K) K_R^s + (\alpha_{PU}^K \delta_U^K + \tau_U^K) K_U^s - (2\tau_P^K + \delta_P^K) K_P^s \\
&\quad + \mu_H N_P^K + \gamma_{si}^K K_P^i - \Big(\frac{\beta_{KD} D_P^i}{N_P^K} + \tau + d_H \Big) K_P^s \\
\dot{K}_P^e &= (\alpha_{PR}^K \delta_R^K + \tau_R^K) K_R^e + (\alpha_{PU}^K \delta_U^K + \tau_U^K) K_U^e - (2\tau_P^K + \delta_P^K) K_P^e \\
&\quad + \frac{\beta_{KD} D_P^i}{N_P^K} K_P^s - \Big(\gamma_{ie}^K + \tau + d_H \Big) K_P^e \\
\dot{K}_P^i &= (\alpha_{PR}^K \delta_R^K + \tau_R^K) K_R^i + (\alpha_{PU}^K \delta_U^K + \tau_U^K) K_U^i - (2\tau_P^K + \delta_P^K) K_P^i \\
&\quad + \gamma_{ie}^K K_P^e - \Big(\gamma_{si}^K + d_i^K + \tau + d_H \Big) K_P^i \\
\dot{K}_P^d &= (\alpha_{PR}^K \delta_R^K + \tau_R^K) K_R^d + (\alpha_{PU}^K \delta_U^K + \tau_U^K) K_U^d - (2\tau_P^K + \delta_P^K) K_P^d \\
&\quad + d_i^K K_P^i \\
\dot{K}_U^s &= (\alpha_{UP}^K \delta_P^K + \tau_P^K) K_P^s + (\alpha_{UR}^K \delta_R^K + \tau_R^K) K_R^s - (2\tau_U^K + \delta_U^K) K_U^s \\
&\quad + \mu_H N_U^K + \gamma_{si}^K K_U^i - \Big(\frac{\beta_{KD} D_U^i}{N_U^K} + \tau + d_H \Big) K_U^s \\
\dot{K}_U^e &= (\alpha_{UP}^K \delta_P^K + \tau_P^K) K_P^e + (\alpha_{UR}^K \delta_R^K + \tau_R^K) K_R^e - (2\tau_U^K + \delta_U^K) K_U^e \\
&\quad + \frac{\beta_{KD} D_U^i}{N_U^K} K_U^s - \Big(\gamma_{ie}^K + \tau + d_H \Big) K_U^e \\
\dot{K}_U^i &= (\alpha_{UP}^K \delta_P^K + \tau_P^K) K_P^i + (\alpha_{UR}^K \delta_R^K + \tau_R^K) K_R^i - (2\tau_U^K + \delta_U^K) K_U^i \\
&\quad + \gamma_{ie}^K K_U^e - \Big(\gamma_{si}^K + d_i^K + \tau + d_H \Big) K_U^i \\
\dot{K}_U^d &= (\alpha_{UP}^K \delta_P^K + \tau_P^K) K_P^d + (\alpha_{UR}^K \delta_R^K + \tau_R^K) K_R^d - (2\tau_U^K + \delta_U^K) K_U^d \\
&\quad + d_i^K K_U^i \\
\dot{K}_R^s &= (\alpha_{RP}^K \delta_P^K + \tau_P^K) K_P^s + (\alpha_{RU}^K \delta_U^K + \tau_U^K) K_U^s - (2\tau_R^K + \delta_R^K) K_R^s \\
&\quad + \mu_H N_R^K + \gamma_{si}^K K_R^i - \Big(\frac{\beta_{KD} D_R^i}{N_R^K} + \tau + d_H \Big) K_R^s \\
\dot{K}_R^e &= (\alpha_{RP}^K \delta_P^K + \tau_P^K) K_P^e + (\alpha_{RU}^K \delta_U^K + \tau_U^K) K_U^e - (2\tau_R^K + \delta_R^K) K_R^e \\
&\quad + \frac{\beta_{KD} D_R^i}{N_R^K} K_R^s - \Big(\gamma_{ie}^K + \tau + d_H \Big) K_R^e \\
\dot{K}_R^i &= (\alpha_{RP}^K \delta_P^K + \tau_P^K) K_P^i + (\alpha_{RU}^K \delta_U^K + \tau_U^K) K_U^i - (2\tau_R^K + \delta_R^K) K_R^i \\
&\quad + \gamma_{ie}^K K_R^e - \Big(\gamma_{si}^K + d_i^K + \tau + d_H \Big) K_R^i
\end{aligned}
$$

$$\dot{K}_R^d = (\alpha_{RP}^K \delta_P^K + \tau_P^K) K_P^d + (\alpha_{RU}^K \delta_U^K + \tau_U^K) K_U^d - (2\tau_R^K + \delta_R^K) K_R^d + d_i^K K_R^i$$

$$\dot{A}_P^s = (\alpha_{PR}^A \delta_R^A + \tau_R^A) A_R^s + (\alpha_{PU}^A \delta_U^A + \tau_U^A) A_U^s - (2\tau_P^A + \delta_P^A) A_P^s + \tau K_P^s + \gamma_{si}^A A_P^i - \Big(\frac{\beta_{AD} D_P^i}{N_P^A} + d_H\Big) A_P^s$$

$$\dot{A}_P^e = (\alpha_{PR}^A \delta_R^A + \tau_R^A) A_R^e + (\alpha_{PU}^A \delta_U^A + \tau_U^A) A_U^e - (2\tau_P^A + \delta_P^A) A_P^e + \frac{\beta_{AD} D_P^i}{N_P^A} A_P^s + \tau K_P^e - \Big(\gamma_{ie}^A + d_H\Big) A_P^e$$

$$\dot{A}_P^i = (\alpha_{PR}^A \delta_R^A + \tau_R^A) A_R^i + (\alpha_{PU}^A \delta_U^A + \tau_U^A) A_U^i - (2\tau_P^A + \delta_P^A) A_P^i + \gamma_{ie}^A A_P^e + \tau K_P^i - \Big(\gamma_{si}^A + d_i^A + d_H\Big) A_P^i$$

$$\dot{A}_P^d = (\alpha_{PR}^A \delta_R^A + \tau_R^A) A_R^d + (\alpha_{PU}^A \delta_U^A + \tau_U^A) A_U^d - (2\tau_P^A + \delta_P^A) A_P^d + d_i^A A_P^i$$

$$\dot{A}_U^s = (\alpha_{UP}^A \delta_P^A + \tau_P^A) A_P^s + (\alpha_{UR}^A \delta_R^A + \tau_R^A) A_R^s - (2\tau_U^A + \delta_U^A) A_U^s + \tau K_U^s + \gamma_{si}^A A_U^i - \Big(\frac{\beta_{AD} D_U^i}{N_U^A} + d_H\Big) A_U^s$$

$$\dot{A}_U^e = (\alpha_{UP}^A \delta_P^A + \tau_P^A) A_P^e + (\alpha_{UR}^A \delta_R^A + \tau_R^A) A_R^e - (2\tau_U^A + \delta_U^A) A_U^e + \frac{\beta_{AD} D_U^i}{N_U^A} A_U^s + \tau K_U^e - \Big(\gamma_{ie}^A + d_H\Big) A_U^e$$

$$\dot{A}_U^i = (\alpha_{UP}^A \delta_P^A + \tau_P^A) A_P^i + (\alpha_{UR}^A \delta_R^A + \tau_R^A) A_R^i - (2\tau_U^A + \delta_U^A) A_U^i + \gamma_{ie}^A A_U^e + \tau K_U^i - \Big(\gamma_{si}^A + d_i^A + d_H\Big) A_U^i$$

$$\dot{A}_U^d = (\alpha_{UP}^A \delta_P^A + \tau_P^A) A_P^d + (\alpha_{UR}^A \delta_R^A + \tau_R^A) A_R^d - (2\tau_U^A + \delta_U^A) A_U^d + d_i^A A_U^i$$

$$\dot{A}_R^s = (\alpha_{RP}^A \delta_P^A + \tau_P^A) A_P^s + (\alpha_{RU}^A \delta_U^A + \tau_U^A) A_U^s - (2\tau_R^A + \delta_R^A) A_R^s + \tau K_R^s + \gamma_{si}^A A_R^i - \Big(\frac{\beta_{AD} D_R^i}{N_R^A} + d_H\Big) A_R^s$$

$$\dot{A}_R^e = (\alpha_{RP}^A \delta_P^A + \tau_P^A) A_P^e + (\alpha_{RU}^A \delta_U^A + \tau_U^A) A_U^e - (2\tau_R^A + \delta_R^A) A_R^e + \frac{\beta_{AD} D_R^i}{N_R^A} A_R^s + \tau K_R^e - \Big(\gamma_{ie}^A + d_H\Big) A_R^e$$

$$
\begin{aligned}
\dot{A}_R^i &= (\alpha_{RP}^A \delta_P^A + \tau_P^A) A_P^i + (\alpha_{RU}^A \delta_U^A + \tau_U^A) A_U^i - (2\tau_R^A + \delta_R^A) A_R^i \\
&\quad + \gamma_{ie}^A A_R^e + \tau K_R^i - \Big(\gamma_{si}^A + d_i^A + d_H\Big) A_R^i \\
\dot{A}_R^d &= (\alpha_{RP}^A \delta_P^A + \tau_P^A) A_P^d + (\alpha_{RU}^A \delta_U^A + \tau_U^A) A_U^d - (2\tau_R^A + \delta_R^A) A_R^d \\
&\quad + d_i^A A_R^i
\end{aligned}
$$
