# Supplementary material for: The role of host mobility in the transmission and spread of Echinococcus granulosus: A Chile-based mathematical modeling approach
Source: PLoS Negl Trop Dis. 2025 Apr 14;19(4):e0012948. doi: 10.1371/journal.pntd.0012948 (PMC11996221; doi:10.1371/journal.pntd.0012948)
Supplement: S2 Appendix — (PDF) [file pntd.0012948.s002.pdf]

S2 Appendix

R_0 for proposed mathematical model

The next-generation reproduction number is given by

$$R_0 = \rho(FV^{-1}),$$

where $\rho(\cdot)$ denotes the spectral radius of FV^{-1}.

$$F = \begin{bmatrix}
0 & 0 & 0 & 0 & 0 & 0 & 0 & 0 & f_{1,9} & 0 & 0 & 0 & 0 & 0 & 0 & 0 & 0 & 0 & 0 \\
0 & 0 & 0 & 0 & 0 & 0 & 0 & 0 & 0 & 0 & f_{2,11} & 0 & 0 & 0 & 0 & 0 & 0 & 0 & 0 \\
0 & 0 & 0 & 0 & 0 & 0 & 0 & 0 & f_{3,9} & 0 & 0 & 0 & 0 & 0 & 0 & 0 & 0 & 0 & 0 \\
0 & 0 & 0 & 0 & 0 & 0 & 0 & 0 & 0 & f_{4,10} & 0 & 0 & 0 & 0 & 0 & 0 & 0 & 0 & 0 \\
0 & 0 & 0 & 0 & 0 & 0 & 0 & 0 & 0 & 0 & f_{5,11} & 0 & 0 & 0 & 0 & 0 & 0 & 0 & 0 \\
0 & 0 & 0 & 0 & 0 & 0 & 0 & 0 & f_{6,9} & 0 & 0 & 0 & 0 & 0 & 0 & 0 & 0 & 0 & 0 \\
0 & 0 & 0 & 0 & 0 & 0 & 0 & 0 & 0 & f_{7,10} & 0 & 0 & 0 & 0 & 0 & 0 & 0 & 0 & 0 \\
0 & 0 & 0 & 0 & 0 & 0 & 0 & 0 & 0 & 0 & f_{8,11} & 0 & 0 & 0 & 0 & 0 & 0 & 0 & 0 \\
0 & 0 & 0 & 0 & 0 & 0 & 0 & 0 & 0 & 0 & 0 & f_{9,12} & f_{9,13} & 0 & 0 & 0 & 0 & 0 & 0 \\
0 & 0 & 0 & 0 & 0 & 0 & 0 & 0 & 0 & 0 & 0 & f_{10,12} & f_{10,13} & 0 & 0 & 0 & 0 & 0 & 0 \\
0 & 0 & 0 & 0 & 0 & 0 & 0 & 0 & 0 & 0 & 0 & f_{11,12} & f_{11,13} & 0 & 0 & 0 & 0 & 0 & 0 \\
0 & 0 & 0 & 0 & 0 & 0 & 0 & 0 & 0 & 0 & 0 & 0 & 0 & 0 & 0 & 0 & 0 & 0 & 0 \\
0 & 0 & 0 & 0 & 0 & 0 & 0 & 0 & 0 & 0 & 0 & 0 & 0 & 0 & 0 & 0 & 0 & 0 & 0 \\
0 & 0 & 0 & 0 & 0 & 0 & 0 & 0 & 0 & 0 & 0 & 0 & 0 & 0 & 0 & 0 & 0 & 0 & 0 \\
0 & 0 & 0 & 0 & 0 & 0 & 0 & 0 & 0 & 0 & 0 & 0 & 0 & 0 & 0 & 0 & 0 & 0 & 0 \\
0 & 0 & 0 & 0 & 0 & 0 & 0 & 0 & 0 & 0 & 0 & 0 & 0 & 0 & 0 & 0 & 0 & 0 & 0 \\
0 & 0 & 0 & 0 & 0 & 0 & 0 & 0 & 0 & 0 & 0 & 0 & 0 & 0 & 0 & 0 & 0 & 0 & 0 \\
0 & 0 & 0 & 0 & 0 & 0 & 0 & 0 & 0 & 0 & 0 & 0 & 0 & 0 & 0 & 0 & 0 & 0 & 0 \\
0 & 0 & 0 & 0 & 0 & 0 & 0 & 0 & 0 & 0 & 0 & 0 & 0 & 0 & 0 & 0 & 0 & 0 & 0
\end{bmatrix}$$

where: $f_{1,9} := \beta_{OD} O_P^{s*}/N_P^O$, $f_{2,11} := \beta_{OD} O_R^{s*}/N_R^O$, $f_{3,9} := \beta_{KD} K_P^{s*}/N_P^K$, $f_{4,10} := \beta_{KD} K_U^{s*}/N_U^K$,
$f_{5,11} := \beta_{KD} K_R^{s*}/N_R^K$, $f_{6,9} := \beta_{AD} A_P^{s*}/N_P^A$, $f_{7,10} := \beta_{AD} A_U^{s*}/N_U^A$, $f_{8,11} := \beta_{AD} A_R^{s*}/N_R^A$, $f_{9,12} := \beta_{DO} d_O D_P^{s*}/N_P^D$, $f_{9,13} := \beta_{DO} d_O D_P^{s*}/N_P^D$, $f_{10,12} := \beta_{DO} d_O D_U^{s*}/N_U^D$, $f_{10,13} := \beta_{DO} d_O D_U^{s*}/N_U^D$, $f_{11,12} := \beta_{DO} d_O D_R^{s*}/N_R^D$, $f_{11,13} := \beta_{DO} d_O D_R^{s*}/N_R^D$.

$$V = \begin{bmatrix}
v_1 & v_2 & 0 & 0 & 0 & 0 & 0 & 0 & 0 & 0 & 0 & 0 & 0 & 0 & 0 & 0 & 0 & 0 & 0 \\
v_3 & v_4 & 0 & 0 & 0 & 0 & 0 & 0 & 0 & 0 & 0 & 0 & 0 & 0 & 0 & 0 & 0 & 0 & 0 \\
0 & 0 & v_5 & v_6 & v_7 & 0 & 0 & 0 & 0 & 0 & 0 & 0 & 0 & 0 & 0 & 0 & 0 & 0 & 0 \\
0 & 0 & v_8 & v_9 & v_{10} & 0 & 0 & 0 & 0 & 0 & 0 & 0 & 0 & 0 & 0 & 0 & 0 & 0 & 0 \\
0 & 0 & v_{11} & v_{12} & v_{13} & 0 & 0 & 0 & 0 & 0 & 0 & 0 & 0 & 0 & 0 & 0 & 0 & 0 & 0 \\
0 & 0 & v_{14} & 0 & 0 & v_{15} & v_{16} & v_{17} & 0 & 0 & 0 & 0 & 0 & 0 & 0 & 0 & 0 & 0 & 0 \\
0 & 0 & 0 & v_{18} & 0 & v_{19} & v_{20} & v_{21} & 0 & 0 & 0 & 0 & 0 & 0 & 0 & 0 & 0 & 0 & 0 \\
0 & 0 & 0 & 0 & v_{22} & v_{23} & v_{24} & v_{25} & 0 & 0 & 0 & 0 & 0 & 0 & 0 & 0 & 0 & 0 & 0 \\
0 & 0 & 0 & 0 & 0 & 0 & 0 & 0 & v_{26} & v_{27} & v_{28} & 0 & 0 & 0 & 0 & 0 & 0 & 0 & 0 \\
0 & 0 & 0 & 0 & 0 & 0 & 0 & 0 & v_{29} & v_{30} & v_{31} & 0 & 0 & 0 & 0 & 0 & 0 & 0 & 0 \\
0 & 0 & 0 & 0 & 0 & 0 & 0 & 0 & v_{32} & v_{33} & v_{34} & 0 & 0 & 0 & 0 & 0 & 0 & 0 & 0 \\
v_{35} & 0 & 0 & 0 & 0 & 0 & 0 & 0 & 0 & 0 & 0 & v_{36} & v_{37} & 0 & 0 & 0 & 0 & 0 & 0 \\
0 & v_{38} & 0 & 0 & 0 & 0 & 0 & 0 & 0 & 0 & 0 & v_{39} & v_{40} & 0 & 0 & 0 & 0 & 0 & 0 \\
0 & 0 & v_{41} & 0 & 0 & 0 & 0 & 0 & 0 & 0 & 0 & 0 & 0 & v_{42} & v_{43} & v_{44} & 0 & 0 & 0 \\
0 & 0 & 0 & v_{45} & 0 & 0 & 0 & 0 & 0 & 0 & 0 & 0 & 0 & v_{46} & v_{47} & v_{48} & 0 & 0 & 0 \\
0 & 0 & 0 & 0 & v_{49} & 0 & 0 & 0 & 0 & 0 & 0 & 0 & 0 & v_{50} & v_{51} & v_{52} & 0 & 0 & 0 \\
0 & 0 & 0 & 0 & 0 & v_{53} & 0 & 0 & 0 & 0 & 0 & 0 & 0 & v_{54} & 0 & 0 & v_{55} & v_{56} & v_{57} \\
0 & 0 & 0 & 0 & 0 & 0 & v_{58} & 0 & 0 & 0 & 0 & 0 & 0 & 0 & v_{59} & 0 & v_{60} & v_{61} & v_{62} \\
0 & 0 & 0 & 0 & 0 & 0 & 0 & v_{63} & 0 & 0 & 0 & 0 & 0 & 0 & 0 & v_{64} & v_{65} & v_{66} & v_{67}
\end{bmatrix}$$

where: $v_1 := \tau_P^O + \gamma_{ie}^O + d_O$, $v_2 := -\alpha_{PR}^O \delta_R^O$, $v_3 := -\tau_P^O$, $v_4 := \alpha_{PR}^O \delta_R^O + \gamma_{ie}^O + d_O$, $v_5 := \gamma_{ie}^K + \tau + d_H + 2\tau_P^K + \delta_P^K$, $v_6 := -(\alpha_{PU}^K \delta_U^K + \tau_U^K)$, $v_7 := -(\alpha_{PR}^K \delta_R^K + \tau_R^K)$, $v_8 := -(\alpha_{UP}^K \delta_P^K + \tau_P^K)$, $v_9 := \gamma_{ie}^K + \tau + d_H + 2\tau_U^K + \delta_U^K$, $v_{10} := -(\alpha_{UR}^K \delta_R^K + \tau_R^K)$, $v_{11} := -(\alpha_{RP}^K \delta_P^K + \tau_P^K)$, $v_{12} := -(\alpha_{RU}^K \delta_U^K + \tau_U^K), v_{13} := \gamma_{ie}^K + \tau + d_H + 2\tau_R^K + \delta_R^K$, $v_{14} := -\tau$, $v_{15} := 2\tau_P^A + \delta_P^A + \gamma_{ie}^A + +d_H, v_{16} := -(\alpha_{PU}^A \delta_U^A + \tau_U^A), v_{17} := -(\alpha_{PR}^A \delta_R^A + \tau_R^A)$, $v_{18} := -\tau$, $v_{19} := -(\alpha_{UP}^A \delta_P^A + \tau_P^A)$, $v_{20} := \gamma_{ie}^A + d_H + 2\tau_U^A + \delta_U^A$, $v_{21} := -(\alpha_{UR}^A \delta_R^A + \tau_R^A)$, $v_{22} := -\tau$, $v_{23} := -(\alpha_{RP}^A \delta_P^A + \tau_P^A)$, $v_{24} := -(\alpha_{RU}^A \delta_U^A + \tau_U^A)$, $v_{25} := \gamma_{ie}^A + d_H + 2\tau_R^A + \delta_R^A$, $v_{26} := 2\tau_P^D + \delta_P^D + d_D + \gamma_{si}^D$, $v_{27} := -(\alpha_{PU}^D \delta_U^D + \tau_U^D)$, $v_{28} := -(\alpha_{PR}^D \delta_R^D + \tau_R^D)$, $v_{29} := -(\alpha_{UP}^D \delta_P^D + \tau_P^D)$, $v_{30} := d_D + \gamma_{si}^D + 2\tau_P^D + \delta_U^D$, $v_{31} := -(\alpha_{UR}^D \delta_R^D + \tau_R^D)$, $v_{32} := -(\alpha_{RP}^D \delta_P^D + \tau_P^D)$, $v_{33} := -(\alpha_{RU}^D \delta_U^D + \tau_U^D)$, $v_{34} := d_D + \gamma_{si}^D + 2\tau_R^D + \delta_R^D$, $v_{35} := -\gamma_{ie}^O$, $v_{36} := \tau_P^O + d_O$, $v_{37} := -\alpha_{PR}^O \delta_R^O$, $v_{38} := -\gamma_{ie}^O$, $v_{39} := -\tau_P^O$, $v_{40} := \alpha_{PR}^O \delta_R^O + d_O$, $v_{41} := -\gamma_{ie}^K$, $v_{42} := \gamma_{si}^K + d_i^K + \tau + d_H + 2\tau_P^K + \delta_P^K$, $v_{43} = -(\alpha_{PU}^K \delta_U^K + \tau_U^K)$, $v_{44} := -(\alpha_{PR}^K \delta_R^K + \tau_R^K)$, $v_{45} := -\gamma_{ie}^K$, $v_{46} := -(\alpha_{UP}^K \delta_P^K + \tau_P^K)$, $v_{47} := 2\tau_U^K + \delta_U^K + \gamma_{si}^K + d_i^K + \tau + d_H$, $v_{48} := -(\alpha_{UR}^K \delta_R^K + \tau_R^K)$, $v_{49} := -\gamma_{ie}^K$, $v_{50} := -(\alpha_{RP}^K \delta_P^K + \tau_P^K)$, $v_{51} := -(\alpha_{RU}^K \delta_U^K + \tau_U^K)$, $v_{52} := 2\tau_R^K + \delta_R^K + \gamma_{si}^K + d_i^K + \tau + d_H$, $v_{53} := -\gamma_{ie}^A$, $v_{54} := -\tau$, $v_{55} := 2\tau_P^A + \delta_P^A + \gamma_{si}^A + d_i^A + d_H$, $v_{56} := -(\alpha_{PU}^A \delta_U^A + \tau_U^A)$, $v_{57} := -(\alpha_{PR}^A \delta_R^A + \tau_R^A)$, $v_{58} := -\gamma_{ie}^A$, $v_{59} := -\tau$, $v_{60} := -(\alpha_{UP}^A \delta_P^A + \tau_P^A)$, $v_{61} := 2\tau_U^A + \delta_U^A + \gamma_{si}^A + d_i^A + d_H$, $v_{62} := -(\alpha_{UR}^A \delta_R^A + \tau_R^A)$, $v_{63} := -\gamma_{ie}^A$, $v_{64} := -\tau$, $v_{65} := -(\alpha_{RP}^A \delta_P^A + \tau_P^A)$, $v_{66} := -(\alpha_{RU}^A \delta_U^A + \tau_U^A)$, $v_{67} := 2\tau_R^A + \delta_R^A + \gamma_{si}^A + d_i^A + d_H$.

$$
G := FV^{-1} = \begin{bmatrix}
0 & 0 & 0 & 0 & 0 & 0 & 0 & 0 & g_{1,9} & g_{1,10} & g_{1,11} & 0 & 0 & 0 & 0 & 0 & 0 & 0 & 0 \\
0 & 0 & 0 & 0 & 0 & 0 & 0 & 0 & g_{2,9} & g_{2,10} & g_{2,11} & 0 & 0 & 0 & 0 & 0 & 0 & 0 & 0 \\
0 & 0 & 0 & 0 & 0 & 0 & 0 & 0 & g_{3,9} & g_{3,10} & g_{3,11} & 0 & 0 & 0 & 0 & 0 & 0 & 0 & 0 \\
0 & 0 & 0 & 0 & 0 & 0 & 0 & 0 & g_{4,9} & g_{4,10} & g_{4,11} & 0 & 0 & 0 & 0 & 0 & 0 & 0 & 0 \\
0 & 0 & 0 & 0 & 0 & 0 & 0 & 0 & g_{5,9} & g_{5,10} & g_{5,11} & 0 & 0 & 0 & 0 & 0 & 0 & 0 & 0 \\
0 & 0 & 0 & 0 & 0 & 0 & 0 & 0 & g_{6,9} & g_{6,10} & g_{6,11} & 0 & 0 & 0 & 0 & 0 & 0 & 0 & 0 \\
0 & 0 & 0 & 0 & 0 & 0 & 0 & 0 & g_{7,9} & g_{7,10} & g_{7,11} & 0 & 0 & 0 & 0 & 0 & 0 & 0 & 0 \\
0 & 0 & 0 & 0 & 0 & 0 & 0 & 0 & g_{8,9} & g_{8,10} & g_{8,11} & 0 & 0 & 0 & 0 & 0 & 0 & 0 & 0 \\
g_{9,1} & g_{9,2} & 0 & 0 & 0 & 0 & 0 & 0 & 0 & 0 & 0 & g_{9,12} & g_{9,13} & 0 & 0 & 0 & 0 & 0 & 0 \\
g_{10,1} & g_{10,2} & 0 & 0 & 0 & 0 & 0 & 0 & 0 & 0 & 0 & g_{10,12} & g_{10,13} & 0 & 0 & 0 & 0 & 0 & 0 \\
g_{11,1} & g_{11,2} & 0 & 0 & 0 & 0 & 0 & 0 & 0 & 0 & 0 & g_{11,12} & g_{11,13} & 0 & 0 & 0 & 0 & 0 & 0 \\
0 & 0 & 0 & 0 & 0 & 0 & 0 & 0 & 0 & 0 & 0 & 0 & 0 & 0 & 0 & 0 & 0 & 0 & 0 \\
0 & 0 & 0 & 0 & 0 & 0 & 0 & 0 & 0 & 0 & 0 & 0 & 0 & 0 & 0 & 0 & 0 & 0 & 0 \\
0 & 0 & 0 & 0 & 0 & 0 & 0 & 0 & 0 & 0 & 0 & 0 & 0 & 0 & 0 & 0 & 0 & 0 & 0 \\
0 & 0 & 0 & 0 & 0 & 0 & 0 & 0 & 0 & 0 & 0 & 0 & 0 & 0 & 0 & 0 & 0 & 0 & 0 \\
0 & 0 & 0 & 0 & 0 & 0 & 0 & 0 & 0 & 0 & 0 & 0 & 0 & 0 & 0 & 0 & 0 & 0 & 0 \\
0 & 0 & 0 & 0 & 0 & 0 & 0 & 0 & 0 & 0 & 0 & 0 & 0 & 0 & 0 & 0 & 0 & 0 & 0 \\
0 & 0 & 0 & 0 & 0 & 0 & 0 & 0 & 0 & 0 & 0 & 0 & 0 & 0 & 0 & 0 & 0 & 0 & 0 \\
0 & 0 & 0 & 0 & 0 & 0 & 0 & 0 & 0 & 0 & 0 & 0 & 0 & 0 & 0 & 0 & 0 & 0 & 0
\end{bmatrix}
$$

where:

$$
\begin{aligned}
g_{9,1} \quad := \quad & \frac{\gamma_{ie}^O \alpha_{PR}^O \delta_R^O \tau_P^O (\beta_{DO} d_O / N_P^D) D_P^{s*}}{(\alpha_{PR}^O \delta_R^O (\gamma_{ie}^O + d_O) + \tau_P^O (\gamma_{ie}^O + d_O) + (\gamma_{ie}^O + d_O)^2)(\alpha_{PR}^O \delta_R^O d_O + \tau_P^O d_O + d_O^2)} \\
+ \quad & \frac{\gamma_{ie}^O \alpha_{PR}^O \delta_R^O (\gamma_{ie}^O + d_O)(\beta_{DO} d_O / N_P^D) D_P^{s*}}{(\alpha_{PR}^O \delta_R^O (\gamma_{ie}^O + d_O) + \tau_P^O (\gamma_{ie}^O + d_O) + (\gamma_{ie}^O + d_O)^2)(\alpha_{PR}^O \delta_R^O d_O + \tau_P^O d_O + d_O^2)} \\
+ \quad & \frac{\gamma_{ie}^O \alpha_{PR}^O \delta_R^O d_O (\beta_{DO} d_O / N_P^D) D_P^{s*}}{(\alpha_{PR}^O \delta_R^O (\gamma_{ie}^O + d_O) + \tau_P^O (\gamma_{ie}^O + d_O) + (\gamma_{ie}^O + d_O)^2)(\alpha_{PR}^O \delta_R^O d_O + \tau_P^O d_O + d_O^2)} \\
+ \quad & \frac{\gamma_{ie}^O (\gamma_{ie}^O + d_O) d_O (\beta_{DO} d_O / N_P^D) D_P^{s*}}{(\alpha_{PR}^O \delta_R^O (\gamma_{ie}^O + d_O) + \tau_P^O (\gamma_{ie}^O + d_O) + (\gamma_{ie}^O + d_O)^2)(\alpha_{PR}^O \delta_R^O d_O + \tau_P^O d_O + d_O^2)} \\
+ \quad & \frac{\gamma_{ie}^O (\alpha_{PR}^O \delta_R^O)^2 (\beta_{DO} d_O / N_P^D) D_P^{s*}}{(\alpha_{PR}^O \delta_R^O (\gamma_{ie}^O + d_O) + \tau_P^O (\gamma_{ie}^O + d_O) + (\gamma_{ie}^O + d_O)^2)(\alpha_{PR}^O \delta_R^O d_O + \tau_P^O d_O + d_O^2)} \\
+ \quad & \frac{\gamma_{ie}^O (\alpha_{PR}^O \delta_R^O + \tau_P^O + \gamma_{ie}^O + 2 d_O) \tau_P^O (\beta_{DO} d_O / N_P^D) D_P^{s*}}{(\alpha_{PR}^O \delta_R^O (\gamma_{ie}^O + d_O) + \tau_P^O (\gamma_{ie}^O + d_O) + (\gamma_{ie}^O + d_O)^2)(\alpha_{PR}^O \delta_R^O d_O + \tau_P^O d_O + d_O^2)}
\end{aligned}
$$

$$
\begin{aligned}
g_{10,1} \quad := \quad & \frac{\gamma_{ie}^O \alpha_{PR}^O \delta_R^O \tau_P^O (\beta_{DO} d_O / N_U^D) D_U^{s*}}{(\alpha_{PR}^O \delta_R^O (\gamma_{ie}^O + d_O) + \tau_P^O (\gamma_{ie}^O + d_O) + (\gamma_{ie}^O + d_O)^2)(\alpha_{PR}^O \delta_R^O d_O + \tau_P^O d_O + d_O^2)} \\
+ \quad & \frac{\gamma_{ie}^O \alpha_{PR}^O \delta_R^O (\gamma_{ie}^O + d_O)(\beta_{DO} d_O / N_U^D) D_U^{s*}}{(\alpha_{PR}^O \delta_R^O (\gamma_{ie}^O + d_O) + \tau_P^O (\gamma_{ie}^O + d_O) + (\gamma_{ie}^O + d_O)^2)(\alpha_{PR}^O \delta_R^O d_O + \tau_P^O d_O + d_O^2)} \\
+ \quad & \frac{\gamma_{ie}^O \alpha_{PR}^O \delta_R^O d_O (\beta_{DO} d_O / N_U^D) D_U^{s*}}{(\alpha_{PR}^O \delta_R^O (\gamma_{ie}^O + d_O) + \tau_P^O (\gamma_{ie}^O + d_O) + (\gamma_{ie}^O + d_O)^2)(\alpha_{PR}^O \delta_R^O d_O + \tau_P^O d_O + d_O^2)} \\
+ \quad & \frac{\gamma_{ie}^O (\gamma_{ie}^O + d_O) d_O (\beta_{DO} d_O / N_U^D) D_U^{s*}}{(\alpha_{PR}^O \delta_R^O (\gamma_{ie}^O + d_O) + \tau_P^O (\gamma_{ie}^O + d_O) + (\gamma_{ie}^O + d_O)^2)(\alpha_{PR}^O \delta_R^O d_O + \tau_P^O d_O + d_O^2)} \\
+ \quad & \frac{\gamma_{ie}^O (\alpha_{PR}^O \delta_R^O)^2 (\beta_{DO} d_O / N_U^D) D_U^{s*}}{(\alpha_{PR}^O \delta_R^O (\gamma_{ie}^O + d_O) + \tau_P^O (\gamma_{ie}^O + d_O) + (\gamma_{ie}^O + d_O)^2)(\alpha_{PR}^O \delta_R^O d_O + \tau_P^O d_O + d_O^2)} \\
+ \quad & \frac{\gamma_{ie}^O (\alpha_{PR}^O \delta_R^O + \tau_P^O + \gamma_{ie}^O + 2 d_O) \tau_P^O (\beta_{DO} d_O / N_U^D) D_U^{s*}}{(\alpha_{PR}^O \delta_R^O (\gamma_{ie}^O + d_O) + \tau_P^O (\gamma_{ie}^O + d_O) + (\gamma_{ie}^O + d_O)^2)(\alpha_{PR}^O \delta_R^O d_O + \tau_P^O d_O + d_O^2)}
\end{aligned}
$$

$$
\begin{aligned}
g_{11,1} \quad := \quad & \frac{\gamma^O_{ie}\alpha^O_{PR}\delta^O_R\tau^O_P(\beta_{DO}d_O/N^D_R)D^{s*}_R}{(\alpha^O_{PR}\delta^O_R(\gamma^O_{ie}+d_O)+\tau^O_P(\gamma^O_{ie}+d_O)+(\gamma^O_{ie}+d_O)^2)(\alpha^O_{PR}\delta^O_R d_O+\tau^O_P d_O+d^2_O)} \\
+ \quad & \frac{\gamma^O_{ie}\alpha^O_{PR}\delta^O_R(\gamma^O_{ie}+d_O)(\beta_{DO}d_O/N^D_R)D^{s*}_R}{(\alpha^O_{PR}\delta^O_R(\gamma^O_{ie}+d_O)+\tau^O_P(\gamma^O_{ie}+d_O)+(\gamma^O_{ie}+d_O)^2)(\alpha^O_{PR}\delta^O_R d_O+\tau^O_P d_O+d^2_O)} \\
+ \quad & \frac{\gamma^O_{ie}\alpha^O_{PR}\delta^O_R d_O(\beta_{DO}d_O/N^D_R)D^{s*}_R}{(\alpha^O_{PR}\delta^O_R(\gamma^O_{ie}+d_O)+\tau^O_P(\gamma^O_{ie}+d_O)+(\gamma^O_{ie}+d_O)^2)(\alpha^O_{PR}\delta^O_R d_O+\tau^O_P d_O+d^2_O)} \\
+ \quad & \frac{\gamma^O_{ie}(\gamma^O_{ie}+d_O)d_O(\beta_{DO}d_O/N^D_R)D^{s*}_R}{(\alpha^O_{PR}\delta^O_R(\gamma^O_{ie}+d_O)+\tau^O_P(\gamma^O_{ie}+d_O)+(\gamma^O_{ie}+d_O)^2)(\alpha^O_{PR}\delta^O_R d_O+\tau^O_P d_O+d^2_O)} \\
+ \quad & \frac{\gamma^O_{ie}(\alpha^O_{PR}\delta^O_R)^2(\beta_{DO}d_O/N^D_R)D^{s*}_R}{(\alpha^O_{PR}\delta^O_R(\gamma^O_{ie}+d_O)+\tau^O_P(\gamma^O_{ie}+d_O)+(\gamma^O_{ie}+d_O)^2)(\alpha^O_{PR}\delta^O_R d_O+\tau^O_P d_O+d^2_O)} \\
+ \quad & \frac{\gamma^O_{ie}(\alpha^O_{PR}\delta^O_R+\tau^O_P+\gamma^O_{ie}+2d_O)\tau^O_P(\beta_{DO}d_O/N^D_R)D^{s*}_R}{(\alpha^O_{PR}\delta^O_R(\gamma^O_{ie}+d_O)+\tau^O_P(\gamma^O_{ie}+d_O)+(\gamma^O_{ie}+d_O)^2)(\alpha^O_{PR}\delta^O_R d_O+\tau^O_P d_O+d^2_O)}
\end{aligned}
$$

$$
\begin{aligned}
g_{9,2} \quad := \quad & \frac{\gamma^O_{ie}\alpha^O_{PR}\delta^O_R\tau^O_P(\beta_{DO}d_O/N^D_P)D^{s*}_P}{(\alpha^O_{PR}\delta^O_R(\gamma^O_{ie}+d_O)+\tau^O_P(\gamma^O_{ie}+d_O)+(\gamma^O_{ie}+d_O)^2)(\alpha^O_{PR}\delta^O_R d_O+\tau^O_P d_O+d^2_O)} \\
+ \quad & \frac{\gamma^O_{ie}\tau^O_P(\gamma^O_{ie}+d_O)(\beta_{DO}d_O/N^D_P)D^{s*}_P}{(\alpha^O_{PR}\delta^O_R(\gamma^O_{ie}+d_O)+\tau^O_P(\gamma^O_{ie}+d_O)+(\gamma^O_{ie}+d_O)^2)(\alpha^O_{PR}\delta^O_R d_O+\tau^O_P d_O+d^2_O)} \\
+ \quad & \frac{\gamma^O_{ie}\tau^O_P d_O(\beta_{DO}d_O/N^D_P)D^{s*}_P}{(\alpha^O_{PR}\delta^O_R(\gamma^O_{ie}+d_O)+\tau^O_P(\gamma^O_{ie}+d_O)+(\gamma^O_{ie}+d_O)^2)(\alpha^O_{PR}\delta^O_R d_O+\tau^O_P d_O+d^2_O)} \\
+ \quad & \frac{\gamma^O_{ie}(\gamma^O_{ie}+d_O)d_O(\beta_{DO}d_O/N^D_P)D^{s*}_P}{(\tau^O_P(\gamma^O_{ie}+d_O)+\tau^O_P(\gamma^O_{ie}+d_O)+(\gamma^O_{ie}+d_O)^2)(\alpha^O_{PR}\delta^O_R d_O+\tau^O_P d_O+d^2_O)} \\
+ \quad & \frac{\gamma^O_{ie}(\tau^O_P)^2(\beta_{DO}d_O/N^D_P)D^{s*}_P}{(\alpha^O_{PR}\delta^O_R(\gamma^O_{ie}+d_O)+\tau^O_P(\gamma^O_{ie}+d_O)+(\gamma^O_{ie}+d_O)^2)(\alpha^O_{PR}\delta^O_R d_O+\tau^O_P d_O+d^2_O)} \\
+ \quad & \frac{\gamma^O_{ie}(\alpha^O_{PR}\delta^O_R+\tau^O_P+\gamma^O_{ie}+2d_O)\alpha^O_{PR}\delta^O_R(\beta_{DO}d_O/N^D_P)D^{s*}_P}{(\alpha^O_{PR}\delta^O_R(\gamma^O_{ie}+d_O)+\tau^O_P(\gamma^O_{ie}+d_O)+(\gamma^O_{ie}+d_O)^2)(\alpha^O_{PR}\delta^O_R d_O+\tau^O_P d_O+d^2_O)}
\end{aligned}
$$

$$
\begin{aligned}
g_{10,2} \quad := \quad & \frac{\gamma_{ie}^O \alpha_{PR}^O \delta_R^O \tau_P^O (\beta_{DO} d_O / N_U^D) D_U^{s*}}{(\alpha_{PR}^O \delta_R^O (\gamma_{ie}^O + d_O) + \tau_P^O (\gamma_{ie}^O + d_O) + (\gamma_{ie}^O + d_O)^2)(\alpha_{PR}^O \delta_R^O d_O + \tau_P^O d_O + d_O^2)} \\
+ \quad & \frac{\gamma_{ie}^O \tau_P^O (\gamma_{ie}^O + d_O)(\beta_{DO} d_O / N_U^D) D_U^{s*}}{(\alpha_{PR}^O \delta_R^O (\gamma_{ie}^O + d_O) + \tau_P^O (\gamma_{ie}^O + d_O) + (\gamma_{ie}^O + d_O)^2)(\alpha_{PR}^O \delta_R^O d_O + \tau_P^O d_O + d_O^2)} \\
+ \quad & \frac{\gamma_{ie}^O \tau_P^O d_O (\beta_{DO} d_O / N_U^D) D_U^{s*}}{(\alpha_{PR}^O \delta_R^O (\gamma_{ie}^O + d_O) + \tau_P^O (\gamma_{ie}^O + d_O) + (\gamma_{ie}^O + d_O)^2)(\alpha_{PR}^O \delta_R^O d_O + \tau_P^O d_O + d_O^2)} \\
+ \quad & \frac{\gamma_{ie}^O (\gamma_{ie}^O + d_O) d_O (\beta_{DO} d_O / N_U^D) D_U^{s*}}{(\tau_P^O (\gamma_{ie}^O + d_O) + \tau_P^O (\gamma_{ie}^O + d_O) + (\gamma_{ie}^O + d_O)^2)(\alpha_{PR}^O \delta_R^O d_O + \tau_P^O d_O + d_O^2)} \\
+ \quad & \frac{\gamma_{ie}^O (\tau_P^O)^2 (\beta_{DO} d_O / N_U^D) D_U^{s*}}{(\alpha_{PR}^O \delta_R^O (\gamma_{ie}^O + d_O) + \tau_P^O (\gamma_{ie}^O + d_O) + (\gamma_{ie}^O + d_O)^2)(\alpha_{PR}^O \delta_R^O d_O + \tau_P^O d_O + d_O^2)} \\
+ \quad & \frac{\gamma_{ie}^O (\alpha_{PR}^O \delta_R^O + \tau_P^O + \gamma_{ie}^O + 2 d_O) \alpha_{PR}^O \delta_R^O (\beta_{DO} d_O / N_U^D) D_U^{s*}}{(\alpha_{PR}^O \delta_R^O (\gamma_{ie}^O + d_O) + \tau_P^O (\gamma_{ie}^O + d_O) + (\gamma_{ie}^O + d_O)^2)(\alpha_{PR}^O \delta_R^O d_O + \tau_P^O d_O + d_O^2)}
\end{aligned}
$$

$$
\begin{aligned}
g_{11,2} \quad := \quad & \frac{\gamma_{ie}^O \alpha_{PR}^O \delta_R^O \tau_P^O (\beta_{DO} d_O / N_R^D) D_R^{s*}}{(\alpha_{PR}^O \delta_R^O (\gamma_{ie}^O + d_O) + \tau_P^O (\gamma_{ie}^O + d_O) + (\gamma_{ie}^O + d_O)^2)(\alpha_{PR}^O \delta_R^O d_O + \tau_P^O d_O + d_O^2)} \\
+ \quad & \frac{\gamma_{ie}^O \tau_P^O (\gamma_{ie}^O + d_O)(\beta_{DO} d_O / N_R^D) D_R^{s*}}{(\alpha_{PR}^O \delta_R^O (\gamma_{ie}^O + d_O) + \tau_P^O (\gamma_{ie}^O + d_O) + (\gamma_{ie}^O + d_O)^2)(\alpha_{PR}^O \delta_R^O d_O + \tau_P^O d_O + d_O^2)} \\
+ \quad & \frac{\gamma_{ie}^O \tau_P^O d_O (\beta_{DO} d_O / N_R^D) D_R^{s*}}{(\alpha_{PR}^O \delta_R^O (\gamma_{ie}^O + d_O) + \tau_P^O (\gamma_{ie}^O + d_O) + (\gamma_{ie}^O + d_O)^2)(\alpha_{PR}^O \delta_R^O d_O + \tau_P^O d_O + d_O^2)} \\
+ \quad & \frac{\gamma_{ie}^O (\gamma_{ie}^O + d_O) d_O (\beta_{DO} d_O / N_R^D) D_R^{s*}}{(\tau_P^O (\gamma_{ie}^O + d_O) + \tau_P^O (\gamma_{ie}^O + d_O) + (\gamma_{ie}^O + d_O)^2)(\alpha_{PR}^O \delta_R^O d_O + \tau_P^O d_O + d_O^2)} \\
+ \quad & \frac{\gamma_{ie}^O (\tau_P^O)^2 (\beta_{DO} d_O / N_R^D) D_R^{s*}}{(\alpha_{PR}^O \delta_R^O (\gamma_{ie}^O + d_O) + \tau_P^O (\gamma_{ie}^O + d_O) + (\gamma_{ie}^O + d_O)^2)(\alpha_{PR}^O \delta_R^O d_O + \tau_P^O d_O + d_O^2)} \\
+ \quad & \frac{\gamma_{ie}^O (\alpha_{PR}^O \delta_R^O + \tau_P^O + \gamma_{ie}^O + 2 d_O) \alpha_{PR}^O \delta_R^O (\beta_{DO} d_O / N_R^D) D_R^{s*}}{(\alpha_{PR}^O \delta_R^O (\gamma_{ie}^O + d_O) + \tau_P^O (\gamma_{ie}^O + d_O) + (\gamma_{ie}^O + d_O)^2)(\alpha_{PR}^O \delta_R^O d_O + \tau_P^O d_O + d_O^2)}
\end{aligned}
$$

$$
\begin{aligned}
g_{1,9} \quad := \quad & \frac{(d_D + \gamma_{si}^D)(2\tau_U^D + \delta_U^D)(\beta_{OD} / N_P^O) O_P^{s*}}{Q_1} \\
+ \quad & \frac{(d_D + \gamma_{si}^D)(2\tau_R^D + \delta_R^D)(\beta_{OD} / N_P^O) O_P^{s*}}{Q_1} \\
- \quad & \frac{(\alpha_{UR}^D \delta_R^D + \tau_R^D)(\alpha_{RU}^D \delta_U^D + \tau_U^D)(\beta_{OD} / N_P^O) O_P^{s*}}{Q_1} \\
+ \quad & \frac{(2\tau_R^D + \delta_R^D)(2\tau_U^D + \delta_U^D)(\beta_{OD} / N_P^O) O_P^{s*}}{Q_1} \\
+ \quad & \frac{(d_D + \gamma_{si}^D)^2 (\beta_{OD} / N_P^O) O_P^{s*}}{Q_1}
\end{aligned}
$$

$$
\begin{aligned}
g_{2,9} \quad &:= \quad \frac{(d_D + \gamma_{si}^D)(\alpha_{RP}^D \delta_P^D + \tau_P^D)(\beta_{OD}/N_R^O)O_R^{s*}}{Q_1} \\
&+ \quad \frac{(\alpha_{UP}^D \delta_P^D + \tau_P^D)(\alpha_{RU}^D \delta_U^D + \tau_U^D)(\beta_{OD}/N_R^O)O_R^{s*}}{Q_1} \\
&+ \quad \frac{(\alpha_{RP}^D \delta_P^D + \tau_P^D)(2\tau_U^D + \delta_U^D)(\beta_{OD}/N_R^O)O_R^{s*}}{Q_1}
\end{aligned}
$$

$$
\begin{aligned}
g_{3,9} \quad &:= \quad \frac{(d_D + \gamma_{si}^D)(2\tau_U^D + \delta_U^D)(\beta_{KD}/N_P^K)K_P^{s*}}{Q_1} \\
&+ \quad \frac{(d_D + \gamma_{si}^D)(2\tau_R^D + \delta_R^D)(\beta_{KD}/N_P^K)K_P^{s*}}{Q_1} \\
&- \quad \frac{(\alpha_{UR}^D \delta_R^D + \tau_R^D)(\alpha_{RU}^D \delta_U^D + \tau_U^D)(\beta_{KD}/N_P^K)K_P^{s*}}{Q_1} \\
&+ \quad \frac{(2\tau_R^D + \delta_R^D)(2\tau_U^D + \delta_U^D)(\beta_{KD}/N_P^K)K_P^{s*}}{Q_1} \\
&+ \quad \frac{(d_D + \gamma_{si}^D)^2(\beta_{KD}/N_P^K)K_P^{s*}}{Q_1}
\end{aligned}
$$

$$
\begin{aligned}
g_{4,9} \quad &:= \quad \frac{(d_D + \gamma_{si}^D)(\alpha_{UP}^D \delta_P^D + \tau_P^D)(\beta_{KD}/N_U^K)K_U^{s*}}{Q_1} \\
&+ \quad \frac{(\alpha_{UR}^D \delta_R^D + \tau_R^D)(\alpha_{RP}^D \delta_P^D + \tau_P^D)(\beta_{KD}/N_U^K)K_U^{s*}}{Q_1} \\
&+ \quad \frac{(2\tau_R^D + \delta_R^D)(\alpha_{UP}^D \delta_P^D + \tau_P^D)(\beta_{KD}/N_U^K)K_U^{s*}}{Q_1}
\end{aligned}
$$

$$
\begin{aligned}
g_{5,9} \quad &:= \quad \frac{(d_D + \gamma_{si}^D)(\alpha_{RP}^D \delta_P^D + \tau_P^D)(\beta_{KD}/N_R^K)K_R^{s*}}{Q_1} \\
&+ \quad \frac{(\alpha_{UP}^D \delta_P^D + \tau_P^D)(\alpha_{RU}^D \delta_U^D + \tau_U^D)(\beta_{KD}/N_R^K)K_R^{s*}}{Q_1} \\
&+ \quad \frac{(\alpha_{RP}^D \delta_P^D + \tau_P^D)(2\tau_U^D + \delta_U^D)(\beta_{KD}/N_R^K)K_R^{s*}}{Q_1}
\end{aligned}
$$

$$
\begin{aligned}
g_{6,9} \quad := \quad & \frac{(d_D+\gamma_{si}^D)(2\tau_U^D+\delta_U^D)(\beta_{AD}/N_P^A)A_P^{s*}}{Q_1} \\
+ \quad & \frac{(d_D+\gamma_{si}^D)(2\tau_R^D+\delta_R^D)(\beta_{AD}/N_P^A)A_P^{s*}}{Q_1} \\
- \quad & \frac{(\alpha_{UR}^D\delta_R^D+\tau_R^D)(\alpha_{RU}^D\delta_U^D+\tau_U^D)(\beta_{AD}/N_P^A)A_P^{s*}}{Q_1} \\
+ \quad & \frac{(2\tau_R^D+\delta_R^D)(2\tau_U^D+\delta_U^D)(\beta_{AD}/N_P^A)A_P^{s*}}{Q_1} \\
+ \quad & \frac{(d_D+\gamma_{si}^D)^2(\beta_{AD}/N_P^A)A_P^{s*}}{Q_1}
\end{aligned}
$$

$$
\begin{aligned}
g_{7,9} \quad := \quad & \frac{(d_D+\gamma_{si}^D)(\alpha_{UP}^D\delta_P^D+\tau_P^D)(\beta_{AD}/N_U^A)A_U^{s*}}{Q_1} \\
+ \quad & \frac{(\alpha_{UR}^D\delta_R^D+\tau_R^D)(\alpha_{RP}^D\delta_P^D+\tau_P^D)(\beta_{AD}/N_U^A)A_U^{s*}}{Q_1} \\
+ \quad & \frac{(2\tau_R^D+\delta_R^D)(\alpha_{UP}^D\delta_P^D+\tau_P^D)(\beta_{AD}/N_U^A)A_U^{s*}}{Q_1}
\end{aligned}
$$

$$
\begin{aligned}
g_{8,9} \quad := \quad & \frac{(d_D+\gamma_{si}^D)(\alpha_{RP}^D\delta_P^D+\tau_P^D)(\beta_{AD}/N_R^A)A_R^{s*}}{Q_1} \\
+ \quad & \frac{(\alpha_{UP}^D\delta_P^D+\tau_P^D)(\alpha_{RU}^D\delta_U^D+\tau_U^D)(\beta_{AD}/N_R^A)A_R^{s*}}{Q_1} \\
+ \quad & \frac{(\alpha_{RP}^D\delta_P^D+\tau_P^D)(2\tau_U^D+\delta_U^D)(\beta_{AD}/N_R^A)A_R^{s*}}{Q_1}
\end{aligned}
$$

$$
\begin{aligned}
Q_1 \quad := \quad & (2\tau_P^D+\delta_P^D)(d_D+\gamma_{si}^D)^2+(d_D+\gamma_{si}^D)^2(2\tau_U^D+\delta_U^D)(d_D+\gamma_{si}^D)^2(2\tau_R^D+\delta_R^D) \\
+ \quad & (d_D+\gamma_{si}^D)^3-(\alpha_{PU}^D\delta_U^D+\tau_U^D)(d_D+\gamma_{si}^D)(\alpha_{UP}^D\delta_P^D+\tau_P^D) \\
- \quad & (\alpha_{PR}^D\delta_R^D+\tau_R^D)(d_D+\gamma_{si}^D)(\alpha_{RP}^D\delta_P^D+\tau_P^D)+(2\tau_P^D+\delta_P D)(d_D+\gamma_{si}^D)(2\tau_U^D+\delta_U^D) \\
- \quad & (\alpha_{PR}^D\delta_R^D+\tau_R^D)(\alpha_{UP}^D\delta_P^D+\tau_P^D)(\alpha_{RU}^D\delta_U^D+\tau_U^D)-(\alpha_{PR}^D\delta_R^D+\tau_R^D)(2\tau_U^D \\
+ \quad & \delta_U^D)(\alpha_{RP}^D\delta_P^D+\tau_P^D)-(\alpha_{PU}^D\delta_U^D+\tau_U^D)(\alpha_{UR}^D\delta_R^D+\tau_R^D)(\alpha_{RP}^D\delta_P^D+\tau_P^D) \\
- \quad & (\alpha_{PU}^D\delta_U^D+\tau_U^D)(\alpha_{UP}^D\delta_P^D+\tau_P^D)(2\tau_R^D+\delta_R^D)+(2\tau_P^D+\delta_P^D)(d_D+\gamma_{si}^D)(2\tau_R^D+\delta_R^D) \\
- \quad & (2\tau_P^D+\delta_P^D)(\alpha_{UR}^D\delta_R^D+\tau_R^D)(\alpha_{RU}^D\delta_U^D+\tau_U^D)+(2\tau_P^D+\delta_P^D)(2\tau_U^D+\delta_U^D)(2\tau_R^D+\delta_R^D) \\
- \quad & (d_D+\gamma_{si}^D)(\alpha_{UR}^D\delta_R^D+\tau_R^D)(\alpha_{RU}^D\delta_U^D+\tau_U^D)+(d_D+\gamma_{si}^D)(2\tau_U^D+\delta_U^D)(2\tau_R^D+\delta_R^D)
\end{aligned}
$$

$$
\begin{aligned}
g_{1,10} \quad := \quad & \frac{(d_D + \gamma_{si}^D)(\alpha_{PU}^D \delta_U^D + \tau_U^D)(\beta_{OD}/N_P^O)O_P^{s*}}{Q_2} \\
+ \quad & \frac{(\alpha_{PR}^D \delta_R^D + \tau_R^D)(\alpha_{RU}^D \delta_U^D + \tau_U^D)(\beta_{OD}/N_P^O)O_P^{s*}}{Q_2} \\
+ \quad & \frac{(\alpha_{PU}^D \delta_U^D + \tau_U^D)(2\tau_R^D + \delta_R^D)(\beta_{OD}/N_P^O)O_P^{s*}}{Q_2}
\end{aligned}
$$

$$
\begin{aligned}
g_{2,10} \quad := \quad & \frac{(\alpha_{RP}^D \delta_P^D + \tau_P^D)(\alpha_{PU}^D \delta_U^D + \tau_U^D)(\beta_{OD}/N_R^O)O_R^{s*}}{Q_2} \\
+ \quad & \frac{(2\tau_P^D + \delta_P^D)(\alpha_{RU}^D \delta_U^D + \tau_U^D)(\beta_{OD}/N_R^O)O_R^{s*}}{Q_2} \\
+ \quad & \frac{(d_D + \gamma_{si}^D)(\alpha_{RU}^D \delta_U^D + \tau_U^D)(\beta_{OD}/N_R^O)O_R^{s*}}{Q_2}
\end{aligned}
$$

$$
\begin{aligned}
g_{3,10} \quad := \quad & \frac{(d_D + \gamma_{si}^D)(\alpha_{PU}^D \delta_U^D + \tau_U^D)(\beta_{KD}/N_P^K)K_P^{s*}}{Q_2} \\
+ \quad & \frac{(\alpha_{PR}^D \delta_R^D + \tau_R^D)(\alpha_{RU}^D \delta_U^D + \tau_U^D)(\beta_{KD}/N_P^K)K_P^{s*}}{Q_2} \\
+ \quad & \frac{(\alpha_{PU}^D \delta_U^D + \tau_U^D)(2\tau_R^D + \delta_R^D)(\beta_{KD}/N_P^K)K_P^{s*}}{Q_2}
\end{aligned}
$$

$$
\begin{aligned}
g_{4,10} \quad := \quad & \frac{(2\tau_P^D + \delta_P^D)(d_D + \gamma_{si}^D)(\beta_{KD}/N_U^K)K_U^{s*}}{Q_2} \\
- \quad & \frac{(\alpha_{PR}^D \delta_R^D + \tau_R^D)(\alpha_{RP}^D \delta_P^D + \tau_P^D)(\beta_{KD}/N_U^K)K_U^{s*}}{Q_2} \\
+ \quad & \frac{(2\tau_P^D + \delta_P^D)(2\tau_R^D + \delta_R^D)(\beta_{KD}/N_U^K)K_U^{s*}}{Q_2} \\
+ \quad & \frac{(d_D + \gamma_{si}^D)(2\tau_R^D + \delta_R^D)(\beta_{KD}/N_U^K)K_U^{s*}}{Q_2} \\
+ \quad & \frac{(d_D + \gamma_{si}^D)^2(\beta_{KD}/N_U^K)K_U^{s*}}{Q_2}
\end{aligned}
$$

$$
\begin{aligned}
g_{5,10} \quad := \quad & \frac{(\alpha_{RP}^D \delta_P^D + \tau_P^D)(\alpha_{PU}^D \delta_U^D + \tau_U^D)(\beta_{KD}/N_R^K)K_R^{s*}}{Q_2} \\
+ \quad & \frac{(2\tau_P^D + \delta_P^D)(\alpha_{RU}^D \delta_U^D + \tau_U^D)(\beta_{KD}/N_R^K)K_R^{s*}}{Q_2} \\
+ \quad & \frac{(d_D + \gamma_{si}^D)(\alpha_{RU}^D \delta_U^D + \tau_U^D)(\beta_{KD}/N_R^K)K_R^{s*}}{Q_2}
\end{aligned}
$$

$$
\begin{aligned}
g_{6,10} \quad := \quad & \frac{(\alpha^D_{RP}\delta^D_P + \tau^D_P)(\alpha^D_{PU}\delta^D_U + \tau^D_U)(\beta_{AD}/N^A_P)A^{s*}_P}{Q_2} \\
+ \quad & \frac{(2\tau^D_P + \delta^D_P)(\alpha^D_{RU}\delta^D_U + \tau^D_U)(\beta_{AD}/N^A_P)A^{s*}_P}{Q_2} \\
+ \quad & \frac{(d_D + \gamma^D_{si})(\alpha^D_{RU}\delta^D_U + \tau^D_U)(\beta_{AD}/N^A_P)A^{s*}_P}{Q_2}
\end{aligned}
$$

$$
\begin{aligned}
g_{7,10} \quad := \quad & \frac{(2\tau^D_P + \delta^D_P)(d_D + \gamma^D_{si})(\beta_{AD}/N^A_U)A^{s*}_U}{Q_2} \\
- \quad & \frac{(\alpha^D_{PR}\delta^D_R + \tau^D_R)(\alpha^D_{RP}\delta^D_P + \tau^D_P)(\beta_{AD}/N^A_U)A^{s*}_U}{Q2} \\
+ \quad & \frac{(2\tau^D_P + \delta^D_P)(2\tau^D_R + \delta^D_R)(\beta_{AD}/N^A_U)A^{s*}_U}{Q_2} \\
+ \quad & \frac{(d_D + \gamma^D_{si})(2\tau^D_R + \delta^D_R)(\beta_{AD}/N^A_U)A^{s*}_U}{Q_2} \\
+ \quad & \frac{(d_D + \gamma^D_{si})^2(\beta_{AD}/N^A_U)A^{s*}_U}{Q_2}
\end{aligned}
$$

$$
\begin{aligned}
g_{8,10} \quad := \quad & \frac{(\alpha^D_{RP}\delta^D_P + \tau^D_P)(\alpha^D_{PU}\delta^D_U + \tau^D_U)(\beta_{AD}/N^A_R)A^{s*}_R}{Q_2} \\
+ \quad & \frac{(2\tau^D_P + \delta^D_P)(\alpha^D_{RU}\delta^D_U + \tau^D_U)(\beta_{AD}/N^A_R)A^{s*}_R}{Q_2} \\
+ \quad & \frac{(d_D + \gamma^D_{si})(\alpha^D_{RU}\delta^D_U + \tau^D_U)(\beta_{AD}/N^A_R)A^{s*}_R}{Q_2}
\end{aligned}
$$

$$
\begin{aligned}
Q_2 \quad := \quad & (2\tau^D_P + \delta^D_P)(d_D + \gamma^D_{si})^2 + (d_D + \gamma^D_{si})^2(2\tau^D_U + \delta^D_U) + (d_D + \gamma^D_{si})^2(2\tau^D_R + \delta^D_R) \\
+ \quad & (d_D + \gamma^D_{si})^3 - (\alpha^D_{PU}\delta^D_U + \tau^D_U)(d_D + \gamma^D_{si})(\alpha^D_{UP}\delta^D_P + \tau^D_P) \\
- \quad & (\alpha^D_{PR}\delta^D_R + \tau^D_R)(d_D + \gamma^D_{si})(\alpha^D_{RP}\delta^D_P + \tau^D_P) + (2\tau^D_P + \delta^D_P)(d_D + \gamma^D_{si})(2\tau^D_U + \delta^D_U) \\
- \quad & (\alpha^D_{PR}\delta^D_R + \tau^D_R)(\alpha^D_{UP}\delta^D_P + \tau^D_P)(\alpha^D_{RU}\delta^D_U + \tau^D_U) - (\alpha^D_{PR}\delta^D_R + \tau^D_R)(2\tau^D_U + \delta^D_U)(\alpha^D_{RP}\delta^D_P + \tau^D_P) \\
- \quad & (\alpha^D_{PU}\delta^D_U + \tau^D_U)(\alpha^D_{UR}\delta^D_R + \tau^D_R)(\alpha^D_{RP}\delta^D_P + \tau^D_P) - (\alpha^D_{PU}\delta^D_U + \tau^D_U)(\alpha^D_{UP}\delta^D_P + \tau^D_P)(2\tau^D_R + \delta^D_R) \\
+ \quad & (2\tau^D_P + \delta^D_P)(d_D + \gamma^D_{si})(2\tau^D_R + \delta^D_R) - (2\tau^D_P + \delta^D_P)(\alpha^D_{UR}\delta^D_R + \tau^D_R)(\alpha^D_{RU}\delta^D_U + \tau^D_U) \\
+ \quad & (2\tau^D_P + \delta^D_P)(2\tau^D_U + \delta^D_U)(2\tau^D_R + \delta^D_R) - (d_D + \gamma^D_{si})(\alpha^D_{UR}\delta^D_R + \tau^D_R)(\alpha^D_{RU}\delta^D_U + \tau^D_U) \\
+ \quad & (d_D + \gamma^D_{si})(2\tau^D_U + \delta^D_U)(2\tau^D_R + \delta^D_R)
\end{aligned}
$$

$$
\begin{aligned}
g_{1,11} \quad &:= \quad \frac{(d_D+\gamma_{si}^D)(\alpha_{PR}^D\delta_R^D+\tau_R^D)(\beta_{OD}/N_P^O)O_P^{s*}}{Q_3} \\
&+ \quad \frac{(\alpha_{PR}^D\delta_R^D+\tau_R^D)(2\tau_U^D+\delta_U^D)(\beta_{OD}/N_P^O)O_P^{s*}}{Q_3} \\
&+ \quad \frac{(\alpha_{PU}^D\delta_U^D+\tau_U^D)(\alpha_{UR}^D\delta_R^D+\tau_R^D)(\beta_{OD}/N_P^O)O_P^{s*}}{Q_3}
\end{aligned}
$$

$$
\begin{aligned}
g_{2,11} \quad &:= \quad \frac{(2\tau_P^D+\delta_P^D)(d_D+\gamma_{si}^D)(\beta_{OD}/N_R^O)O_R^{s*}}{Q_3} \\
&- \quad \frac{(\alpha_{PU}^D\delta_U^D+\tau_U^D)(\alpha_{UP}^D\delta_P^D+\tau_P^D)(\beta_{OD}/N_R^O)O_R^{s*}}{Q_3} \\
&+ \quad \frac{(2\tau_P^D+\delta_P^D)(2\tau_U^D+\delta_U^D)(\beta_{OD}/N_R^O)O_R^{s*}}{Q_3} \\
&+ \quad \frac{(d_D+\gamma_{si}^D)(2\tau_U^D+\delta_U^D)(\beta_{OD}/N_R^O)O_R^{s*}}{Q_3} \\
&+ \quad \frac{(d_D+\gamma_{si}^D)^2(\beta_{OD}/N_R^O)O_R^{s*}}{Q_3}
\end{aligned}
$$

$$
\begin{aligned}
g_{3,11} \quad &:= \quad \frac{(d_D+\gamma_{si}^D)(\alpha_{PR}^D\delta_R^D+\tau_R^D)(\beta_{KD}/N_P^K)K_P^{s*}}{Q_3} \\
&+ \quad \frac{(\alpha_{PR}^D\delta_R^D+\tau_R^D)(2\tau_U^D+\delta_U^D)(\beta_{KD}/N_P^K)K_P^{s*}}{Q_3} \\
&+ \quad \frac{(\alpha_{PU}^D\delta_U^D+\tau_U^D)(\alpha_{UR}^D\delta_R^D+\tau_R^D)(\beta_{KD}/N_P^K)K_P^{s*}}{Q_3}
\end{aligned}
$$

$$
\begin{aligned}
g_{4,11} \quad &:= \quad \frac{(\alpha_{UP}^D\delta_P^D+\tau_P^D)(2\tau_P^D+\delta_P^D)(\beta_{KD}/N_U^K)K_U^{s*}}{Q_3} \\
&+ \quad \frac{(2\tau_P^D+\delta_P^D)(\alpha_{UR}^D\delta_R^D+\tau_R^D)(\beta_{KD}/N_U^K)K_U^{s*}}{Q_3} \\
&+ \quad \frac{(d_D+\gamma_{si}^D)(\alpha_{UR}^D\delta_R^D+\tau_R^D)(\beta_{KD}/N_U^K)K_U^{s*}}{Q_3}
\end{aligned}
$$

$$
\begin{aligned}
g_{5,11} \;:=\; & \frac{(2\tau_P^D + \delta_P^D)(d_D + \gamma_{si}^D)(\beta_{KD}/N_R^K)K_R^{s*}}{Q_3} \\
- \; & \frac{(\alpha_{PU}^D \delta_U^D + \tau_U^D)(\alpha_{UP}^D \delta_P^D + \tau_P^D)(\beta_{KD}/N_R^K)K_R^{s*}}{Q_3} \\
+ \; & \frac{(2\tau_P^D + \delta_P^D)(2\tau_U^D + \delta_U^D)(\beta_{KD}/N_R^K)K_R^{s*}}{Q_3} \\
+ \; & \frac{(d_D + \gamma_{si}^D)(2\tau_U^D + \delta_U^D)(\beta_{KD}/N_R^K)K_R^{s*}}{Q_3} \\
+ \; & \frac{(d_D + \gamma_{si}^D)^2(\beta_{KD}/N_R^K)K_R^{s*}}{Q_3}
\end{aligned}
$$

$$
\begin{aligned}
g_{6,11} \;:=\; & \frac{(d_D + \gamma_{si}^D)(\alpha_{PR}^D \delta_R^D + \tau_R^D)(\beta_{AD}/N_P^A)A_P^{s*}}{Q_3} \\
+ \; & \frac{(\alpha_{PR}^D \delta_R^D + \tau_R^D)(2\tau_U^D + \delta_U^D)(\beta_{AD}/N_P^A)A_P^{s*}}{Q_3} \\
+ \; & \frac{(\alpha_{PU}^D \delta_U^D + \tau_U^D)(\alpha_{UR}^D \delta_R^D + \tau_R^D)(\beta_{AD}/N_P^A)A_P^{s*}}{Q_3}
\end{aligned}
$$

$$
\begin{aligned}
g_{7,11} \;:=\; & \frac{(\alpha_{UP}^D \delta_P^D + \tau_P^D)(2\tau_P^D + \delta_P^D)(\beta_{AD}/N_U^A)A_U^{s*}}{Q_3} \\
+ \; & \frac{(2\tau_P^D + \delta_P^D)(\alpha_{UR}^D \delta_R^D + \tau_R^D)(\beta_{AD}/N_U^A)A_U^{s*}}{Q_3} \\
+ \; & \frac{(d_D + \gamma_{si}^D)(\alpha_{UR}^D \delta_R^D + \tau_R^D)(\beta_{AD}/N_U^A)A_U^{s*}}{Q_3}
\end{aligned}
$$

$$
\begin{aligned}
g_{8,11} \;:=\; & \frac{(2\tau_P^D + \delta_P^D)(d_D + \gamma_{si}^D)(\beta_{AD}/N_R^A)A_R^{s*}}{Q_3} \\
- \; & \frac{(\alpha_{PU}^D \delta_U^D + \tau_U^D)(\alpha_{UP}^D \delta_P^D + \tau_P^D)(\beta_{AD}/N_R^A)A_R^{s*}}{Q_3} \\
+ \; & \frac{(2\tau_P^D + \delta_P^D)(2\tau_U^D + \delta_U^D)(\beta_{AD}/N_R^A)A_R^{s*}}{Q_3} \\
+ \; & \frac{(d_D + \gamma_{si}^D)(2\tau_U^D + \delta_U^D)(\beta_{AD}/N_R^A)A_R^{s*}}{Q_3} \\
+ \; & \frac{(d_D + \gamma_{si}^D)^2(\beta_{AD}/N_R^A)A_R^{s*}}{Q_3}
\end{aligned}
$$

$$
\begin{aligned}
Q_3 \quad := \quad & (2\tau_P^D + \delta_P^D)(d_D + \gamma_{si}^D)^2 + (d_D + \gamma_{si}^D)^2(2\tau_U^D + \delta_U^D) + (d_D + \gamma_{si}^D)^2(2\tau_R^D + \delta_R^D) + (d_D + \gamma_{si}^D)^3 \\
- \quad & (\alpha_{PU}^D \delta_U^D + \tau_U^D)(d_D + \gamma_{si}^D)(\alpha_{UP}^D \delta_P^D + \tau_P^D) - (\alpha_{PR}^D \delta_R^D + \tau_R^D)(d_D + \gamma_{si}^D)(\alpha_{RP}^D \delta_P^D + \tau_P^D) \\
+ \quad & (2\tau_P^D + \delta_P^D)(d_D + \gamma_{si}^D)(2\tau_U^D + \delta_U^D) - (\alpha_{PR}^D \delta_R^D + \tau_R^D)(\alpha_{UP}^D \delta_P^D + \tau_P^D)(\alpha_{RU}^D \delta_U^D + \tau_U^D) \\
- \quad & (\alpha_{PR}^D \delta_R^D + \tau_R^D)(2\tau_U^D + \delta_U^D)(\alpha_{RP}^D \delta_P^D + \tau_P^D) - (\alpha_{PU}^D \delta_U^D + \tau_U^D)(\alpha_{UR}^D \delta_R^D + \tau_R^D)(\alpha_{RP}^D \delta_P^D + \tau_P^D) \\
- \quad & (\alpha_{PU}^D \delta_U^D + \tau_U^D)(\alpha_{UP}^D \delta_P^D + \tau_P^D)(2\tau_R^D + \delta_R^D) + (2\tau_P^D + \delta_P^D)(d_D + \gamma_{si}^D)(2\tau_R^D + \delta_R^D) \\
- \quad & (2\tau_P^D + \delta_P^D)(\alpha_{UR}^D \delta_R^D + \tau_R^D)(\alpha_{RU}^D \delta_U^D + \tau_U^D) + (2\tau_P^D + \delta_P^D)(2\tau_U^D + \delta_U^D)(2\tau_R^D + \delta_R^D) \\
- \quad & (d_D + \gamma_{si}^D)(\alpha_{UR}^D \delta_R^D + \tau_R^D)(\alpha_{RU}^D \delta_U^D + \tau_U^D) + (d_D + \gamma_{si}^D)(2\tau_U^D + \delta_U^D)(2\tau_R^D + \delta_R^D)
\end{aligned}
$$

$$
\begin{aligned}
g_{9,12} \quad &:= \quad \frac{\tau_P^O(\beta_{DO} d_O / N_P^D) D_P^{s*} + (\alpha_{PR}^O \delta_R^O + d_O)(\beta_{DO} d_O / N_P^D) D_P^{s*}}{(\alpha_{PR}^O \delta_R^O d_O + \tau_P^O d_O + d_O^2)} \\
g_{10,12} \quad &:= \quad \frac{\tau_P^O(\beta_{DO} d_O / N_U^D) D_U^{s*} + (\alpha_{PR}^O \delta_R^O + d_O)(\beta_{DO} d_O / N_U^D) D_U^{s*}}{(\alpha_{PR}^O \delta_R^O d_O + \tau_P^O d_O + d_O^2)} \\
g_{11,12} \quad &:= \quad \frac{\tau_P^O(\beta_{DO} d_O / N_R^D) D_R^{s*} + (\alpha_{PR}^O \delta_R^O + d_O)(\beta_{DO} d_O / N_R^D) D_R^{s*}}{(\alpha_{PR}^O \delta_R^O d_O + \tau_P^O d_O + d_O^2)}
\end{aligned}
$$

$$
\begin{aligned}
g_{9,13} \quad &:= \quad \frac{\alpha_{PR}^O \delta_R^O(\beta_{DO} d_O / N_P^D) D_P^{s*} + (\tau_P^O + d_O)(\beta_{DO} d_O / N_P^D) D_P^{s}}{(\alpha_{PR}^O \delta_R^O d_O + \tau_P^O d_O + d_O^2)} \\
g_{10,13} \quad &:= \quad \frac{\alpha_{PR}^O \delta_R^O(\beta_{DO} d_O / N_U^D) D_U^{s*} + (\tau_P^O + d_O)(\beta_{DO} d_O / N_U^D) D_U^{s*}}{(\alpha_{PR}^O \delta_R^O d_O + \tau_P^O d_O + d_O^2)} \\
g_{11,13} \quad &:= \quad \frac{\alpha_{PR}^O \delta_R^O(\beta_{DO} d_O / N_R^D) D_R^{s*} + (\tau_P^O + d_O)(\beta_{DO} d_O / N_R^D) D_R^{s*}}{(\alpha_{PR}^O \delta_R^O d_O + \tau_P^O d_O + d_O^2)}
\end{aligned}
$$

Hence, the reproduction number is given by

$$
R_0 = \frac{(AB)^{1/2}}{C},
$$

where:

$$
\begin{aligned}
A &= ((\gamma_{ie}^{O} + d_O)d_O((d_D + \gamma_{si}^{D})^3 + (2\tau_P^D + \delta_P^D)(d_D + \gamma_{si}^{D})^2 + (d_D + \gamma_{si}^{D})^2(2\tau_U^D\delta_U^D) \\
&+ (d_D + \gamma_{si}^{D})^2(2\tau_R^D + \delta_R^D) - (\alpha_{PU}^D\delta_U^D + \tau_U^D)(d_D + \gamma_{si}^{D})(\alpha_{UP}^D\delta_P^D + \tau_P^D) \\
&- (\alpha_{PR}^D\delta_R^D + \tau_R^D)(d_D + \gamma_{si}^{D})(\alpha_{RP}^D\delta_P^D + \tau_P^D) + (2\tau_P^D + \delta_P^D)(d_D + \gamma_{si}^{D})(2\tau_U^D\delta_U^D) \\
&- (\alpha_{PR}^D\delta_R^D + \tau_R^D)(\alpha_{UP}^D\delta_P^D + \tau_P^D)(\alpha_{RU}^D\delta_U^D + \tau_U^D) - (\alpha_{PU}^D\delta_U^D + \tau_U^D)(\alpha_{UR}^D\delta_R^D + \tau_R^D)(\alpha_{RP}^D\delta_P^D + \tau_P^D) \\
&- (\alpha_{PU}^D\delta_U^D + \tau_U^D)(\alpha_{UP}^D\delta_P^D + \tau_P^D)(2\tau_R^D + \delta_R^D) + (2\tau_P^D + \delta_P^D)(d_D + \gamma_{si}^{D})(2\tau_R^D + \delta_R^D) \\
&- (2\tau_P^D + \delta_P^D)(\alpha_{UR}^D\delta_R^D + \tau_R^D)(\alpha_{RU}^D\delta_U^D + \tau_U^D) + (2\tau_P^D + \delta_P^D)(2\tau_U^D\delta_U^D)(2\tau_R^D + \delta_R^D) \\
&- (d_D + \gamma_{si}^{D})(\alpha_{UR}^D\delta_R^D + \tau_R^D)(\alpha_{RU}^D\delta_U^D + \tau_U^D) + (d_D + \gamma_{si}^{D})(2\tau_U^D\delta_U^D)(2\tau_R^D + \delta_R^D))),
\end{aligned}
$$

$$
\begin{aligned}
B &= (\gamma_{ie}^{O}(D_P^{s*}O_P^{s*}(\beta_{OD}/N_P^O)(\beta_{DO}d_O/N_P^D)((d_D + \gamma_{si}^{D})^2 + (d_D + \gamma_{si}^{D})(2\tau_U^D\delta_U^D) \\
&+ (d_D + \gamma_{si}^{D})(2\tau_R^D + \delta_R^D) - (\alpha_{UR}^D\delta_R^D + \tau_R^D)(\alpha_{RU}^D\delta_U^D + \tau_U^D) \\
&+ (2\tau_U^D\delta_U^D)(2\tau_R^D + \delta_R^D)) + D_R^{s*}O_R^{s*}(\beta_{OD}/N_R^O)(\beta_{DO}d_O/N_R^D)((d_D + \gamma_{si}^{D})^2 \\
&+ (2\tau_P^D + \delta_P^D)(d_D + \gamma_{si}^{D}) + (2\tau_P^D + \delta_P^D)(2\tau_U^D\delta_U^D) - (\alpha_{PU}^D\delta_U^D + \tau_U^D)(\alpha_{UP}^D\delta_P^D + \tau_P^D) \\
&+ (d_D + \gamma_{si}^{D})(2\tau_U^D\delta_U^D)) + D_R^{s*}O_P^{s*}(\beta_{OD}/N_P^O)(\beta_{DO}d_O/N_R^D)((\alpha_{PR}^D\delta_R^D + \tau_R^D)(d_D + \gamma_{si}^{D}) \\
&+ (\alpha_{PR}^D\delta_R^D + \tau_R^D)(2\tau_U^D\delta_U^D) + (\alpha_{PU}^D\delta_U^D + \tau_U^D)(\alpha_{UR}^D\delta_R^D + \tau_R^D)) \\
&+ D_P^{s+}O_R^{s*}(\beta_{OD}/N_R^O)(\beta_{DO}d_O/N_P^D)((d_D + \gamma_{si}^{D})(\alpha_{RP}^D\delta_P^D + \tau_P^D) + (\alpha_{UP}^D\delta_P^D + \tau_P^D)(\alpha_{RU}^D\delta_U^D \\
&+ \tau_U^D) + (2\tau_U^D\delta_U^D)(\alpha_{RP}^D\delta_P^D + \tau_P^D)) + D_U^{s*}O_P^{s*}(\beta_{OD}/N_P^O)((\alpha_{PU}^D\delta_U^D + \tau_U^D)(d_D + \gamma_{si}^{D}) \\
&+ (\alpha_{PR}^D\delta_R^D + \tau_R^D)(\alpha_{RU}^D\delta_U^D + \tau_U^D) + (\alpha_{PU}^D\delta_U^D + \tau_U^D)(2\tau_R^D + \delta_R^D)) \\
&+ D_U^{s*}O_R^{s*}(\beta_{OD}/N_R^O)((\alpha_{PU}^D\delta_U^D + \tau_U^D)(\alpha_{RP}^D\delta_P^D + \tau_P^D) + (2\tau_P^D + \delta_P^D)(\alpha_{RU}^D\delta_U^D + \tau_U^D) \\
&+ (d_D + \gamma_{si}^{D})(\alpha_{RU}^D\delta_U^D + \tau_U^D)))),
\end{aligned}
$$

$$\begin{aligned}
C &= ((\gamma_{ie}^{O} + d_O)d_O((d_D + \gamma_{si}^{D})^3 + (2\tau_P^D + \delta_P^D)(d_D + \gamma_{si}^{D})^2 + (d_D + \gamma_{si}^{D})^2(2\tau_U^D\delta_U^D) \\
&+ (d_D + \gamma_{si}^{D})^2(2\tau_R^D + \delta_R^D) - (\alpha_{PU}^D\delta_U^D + \tau_U^D)(d_D + \gamma_{si}^{D})(\alpha_{UP}^D\delta_P^D + \tau_P^D) \\
&- (\alpha_{PR}^D\delta_R^D + \tau_R^D)(d_D + \gamma_{si}^{D})(\alpha_{RP}^D\delta_P^D + \tau_P^D) + (2\tau_P^D + \delta_P^D)(d_D + \gamma_{si}^{D})(2\tau_U^D\delta_U^D) \\
&- (\alpha_{PR}^D\delta_R^D + \tau_R^D)(\alpha_{UP}^D\delta_P^D + \tau_P^D)(\alpha_{RU}^D\delta_U^D + \tau_U^D) - (\alpha_{PR}^D\delta_R^D + \tau_R^D)(2\tau_U^D\delta_U^D)(\alpha_{RP}^D\delta_P^D + \tau_P^D) \\
&- (\alpha_{PU}^D\delta_U^D + \tau_U^D)(\alpha_{UR}^D\delta_R^D + \tau_R^D)(\alpha_{RP}^D\delta_P^D + \tau_P^D) - (\alpha_{PU}^D\delta_U^D + \tau_U^D)(\alpha_{UP}^D\delta_P^D \\
&+ \tau_P^D)(2\tau_R^D + \delta_R^D) + (2\tau_P^D + \delta_P^D)(d_D + \gamma_{si}^{D})(2\tau_R^D + \delta_R^D) - (2\tau_P^D + \delta_P^D)(\alpha_{UR}^D\delta_R^D \\
&+ \tau_R^D)(\alpha_{RU}^D\delta_U^D + \tau_U^D) + (2\tau_P^D + \delta_P^D)(2\tau_U^D\delta_U^D)(2\tau_R^D + \delta_R^D) - (d_D + \gamma_{si}^{D})(\alpha_{UR}^D\delta_R^D \\
&+ \tau_R^D)(\alpha_{RU}^D\delta_U^D + \tau_U^D) + (d_D + \gamma_{si}^{D})(2\tau_U^D\delta_U^D)(2\tau_R^D + \delta_R^D)))
\end{aligned}$$

and $X_0^* = [0,0,0,0,0,0,0,0,0,0,0,0,0,0,0,0,0,0, D_P^{s*}, D_U^{s*}, D_R^{s*}, O_P^{s*}, O_R^{s*}, K_P^{s*}, K_U^{s*}, K_R^{s*}, A_P^{s*}, A_U^{s*}, A_R^{s*}]$ is the disease-free equilibrium of the model.
