# Supplementary figures and images for: The role of host mobility in the transmission and spread of Echinococcus granulosus: A Chile-based mathematical modeling approach
Source: PLoS Negl Trop Dis. 2025 Apr 14;19(4):e0012948. doi: 10.1371/journal.pntd.0012948 (PMC11996221; doi:10.1371/journal.pntd.0012948)

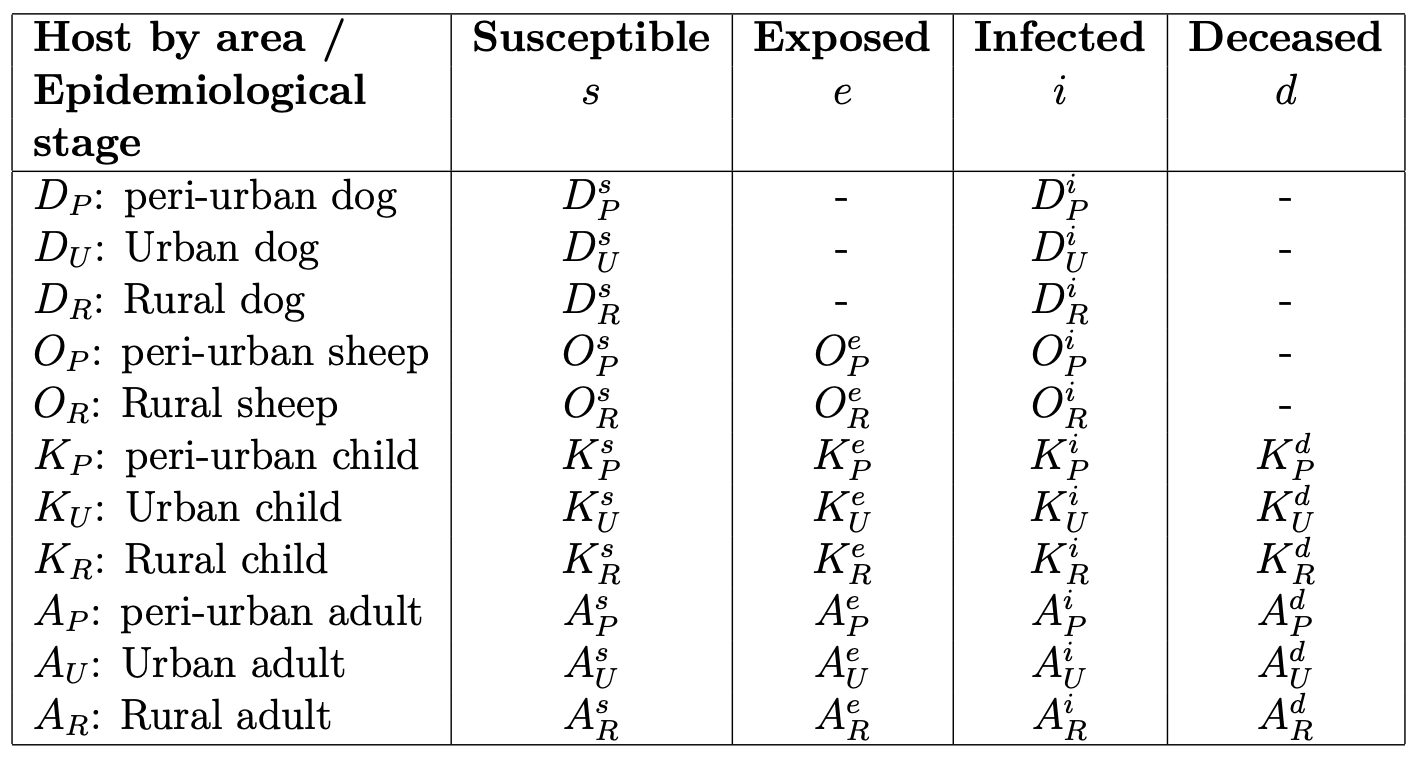

Supplement: S1 Table [file pntd.0012948.s003.png]

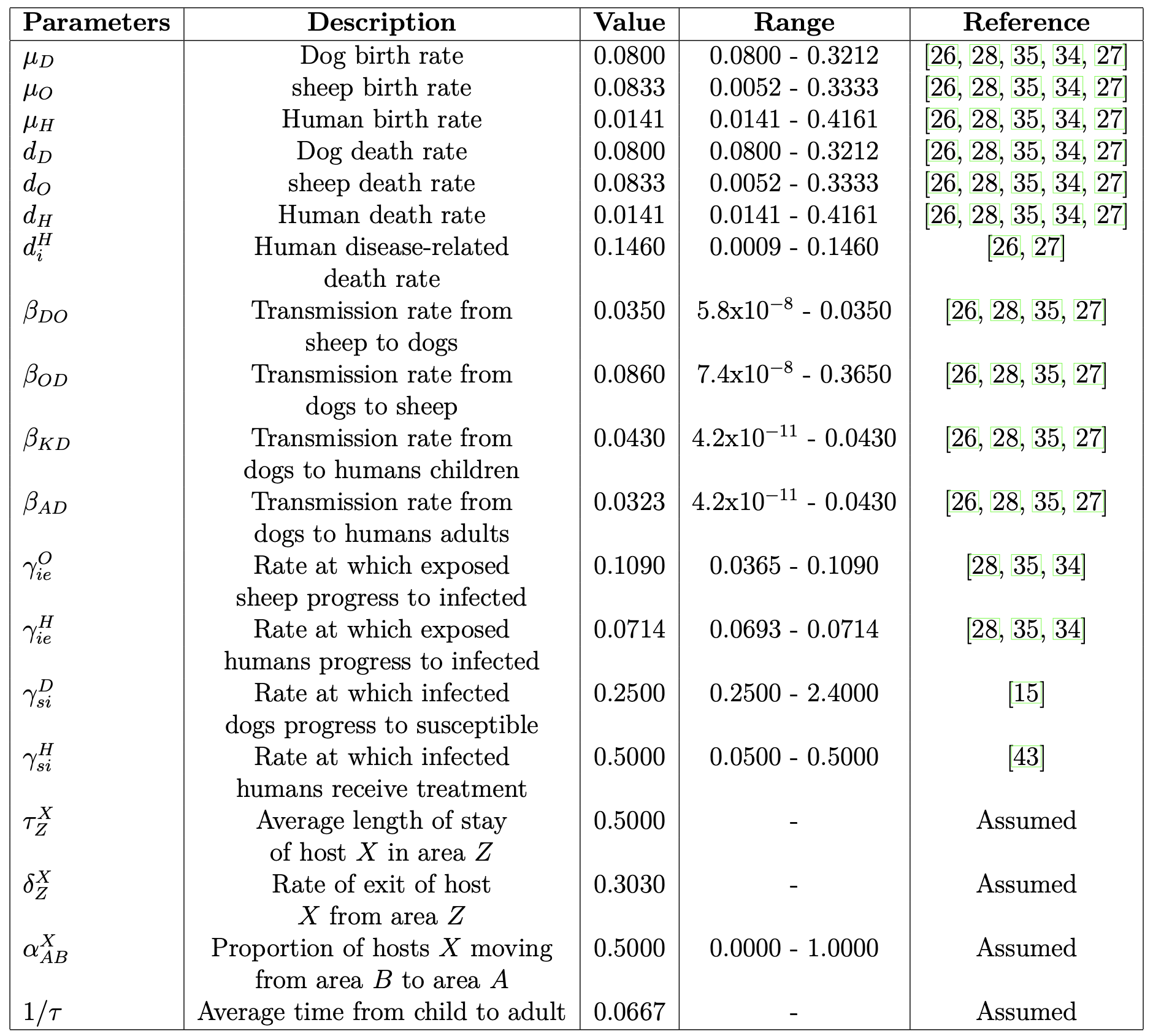

Supplement: S2 Table [file pntd.0012948.s004.png]

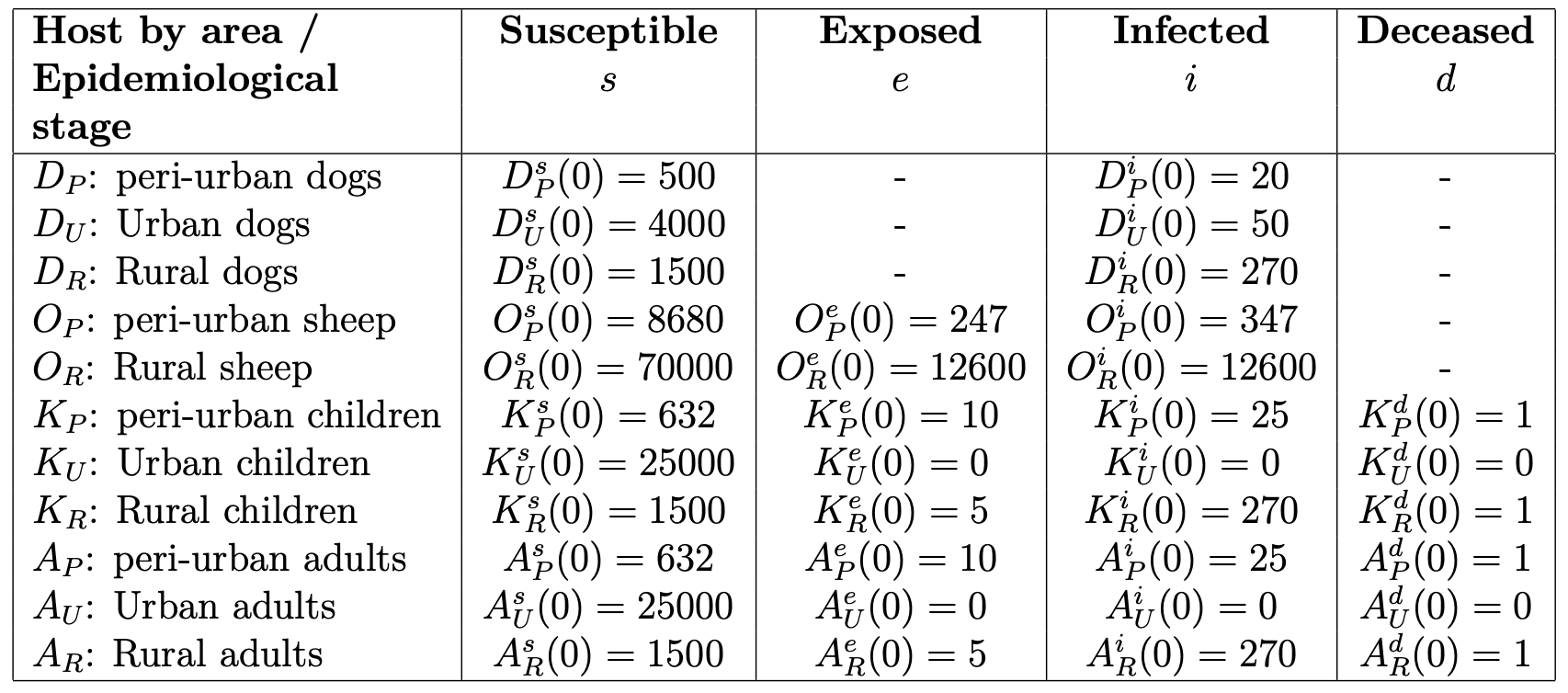

Supplement: S3 Table [file pntd.0012948.s005.png]
